# A conjugal gene drive-like system efficiently suppresses antibiotic resistance in a bacterial population

**DOI:** 10.1038/s44259-026-00181-z

**Published:** 2026-02-02

**Authors:** Saluja Kaduwal, Elizabeth C. Stuart, Ankush Auradkar, Seth Washabaugh, Justin R. Meyer, Ethan Bier

**Affiliations:** 1 Department of Cell and Developmental Biology, University of California, San Diego, La Jolla, CA USA; 2 Tata Institute for Genetics and Society, University of California, San Diego, La Jolla, CA USA; 3 Department of Ecology, Evolution and Behavior, University of California, San Diego, La Jolla, CA USA

**Keywords:** Biotechnology, Microbiology

## Abstract

Antibiotic resistance (AR) is an escalating public health threat, necessitating innovative strategies to control resistant bacterial populations. One promising approach involves engineering genetic elements that can spread within microbial communities to eliminate AR genes. Previously, we developed Pro-Active Genetics (Pro-AG), a CRISPR-based gene-drive-like system capable of reducing AR colony-forming units (CFU) by approximately five logs. Here, we advance this technology by integrating Pro-AG into a conjugative transfer system, enabling efficient dissemination of an anti-AR gene cassette between two bacterial strains. Additionally, we characterize a complementary homology-based deletion (HBD) process, a CRISPR-driven mechanism that precisely removes target DNA sequences flanked by short direct repeats. Our findings reveal that Pro-AG and HBD are differentially influenced by the bacterial RecA pathway and that HBD components can be delivered via plasmids or phages to selectively delete Pro-AG cassettes. This built-in safeguard prevents uncontrolled spread of a gene cassette and mitigates unanticipated side effects. These refinements enhance the efficiency and flexibility of Pro-AG, expanding its potential applications in microbiome engineering, environmental remediation, and clinical interventions aimed at combating antibiotic resistance. More broadly, this work establishes a proof-of-principle for microbiome engineering strategies that could be leveraged to improve health and restore ecological balance.

## Introduction

Antibiotic resistance (AR) poses a serious global health threat and is currently responsible for ~1.27 million deaths worldwide, a toll that is estimated to soar to over 10 million deaths per year by 2050 if left unchecked^1,2^. An important contribution to the increase in AR is the widespread over-prescription of antibiotics, their misuse in animal husbandry, inadequate sewage treatment, and environmental contamination, leading to the emergence and spread of multidrug-resistant bacteria or “superbugs”^3–5^. Bacteria can acquire resistance to antibiotics either by mutation of endogenous chromosomal genes or via exchange of genetic material between bacteria by horizontal gene transfer^6^, the latter being a major route for AR entry into human bacterial pathogens in both environmental and clinical settings. Thus, there is a pressing need for developing new target-specific antimicrobials as well as innovative strategies to combat AR.

One strategy for developing new anti-AR tools is based on the bacterial type II CRISPR (clustered, regularly interspaced, short palindromic repeats) immunity system. A bipartite synthetic system comprised of the *Streptococcus pyogenes* Cas9 (CRISPR-associated protein 9) double-stranded DNA nuclease and a synthetic guide RNA (sgRNA) with a 20-nt targeting sequence^7^ has been broadly used for genome editing in a wide variety of organisms. In prokaryotes, CRISPR tools have been employed for efficient genome editing as well as in developing sequence-specific antimicrobials targeting virulence genes or plasmids conferring antibiotic resistance^8–10^. We previously developed an alternative CRISPR-based strategy referred to as Prokaryotic-Active Genetics (Pro-AG)^11^, which functions efficiently in a self-amplifying fashion similar to that of gene-drive systems developed in diploid eukaryotes^12–16^, or in multi-copy episomal herpesviruses^17^. These Pro-AG systems disrupt genes encoding AR factors carried on a high copy number plasmid by precise insertional inactivation of the AR target gene, outperforming standard cut-and-destroy CRISPR anti-AR approaches by over 100-fold^11^.

The success of the Pro-AG system outlined above in reducing AR by homology-based editing and the recent developments of conjugal transfer systems with a conditional lethal CRISPR system^18–22^ raised the possibility of combining all the required Pro-AG components as a unit into a single conjugal transfer plasmid carrying horizontal transfer machinery for efficient delivery of the Pro-AG system to the population of AR bacteria. Here, we develop and characterize such a second-generation ~65 kb p^Pro-MobV^ plasmid which includes fusion of two key Pro-AG components into a single operon (the three genes encoding the phage-derived λRed recombination proteins plus Cas9), a sgRNA targeting the *bla* resistance gene flanked with homology arms, a broad host-range origin of replication, genes required for plasmid maintenance, stability and conjugation derived from the IncP RK2 conjugative system (referred to collectively as conjugation machinery in our study) as well as an *ori*T site for cis-transfer of these components to recipient bacteria^18,23,24^. Delivery of the p^Pro-MobV^ system from donor cells to trans-conjugated receiver bacteria can efficiently inactivate a *bla* target gene conferring resistance to ampicillin (Amp^R^) carried on a high copy number plasmid, thereby reducing the prevalence of antibiotic resistance by three to five logs depending on the genotype of the receiver bacterial strains. We also identify and characterize a novel Cas9-dependent process to expand the Pro-AG toolkit wherein a sgRNA targets a plasmid for cleavage between two short (25–100 bp) directly repeated sequences. Such cleavage results in efficient and precise deletion of the intervening sequences via homologous recombination, a process we refer to as homology-based deletion (HBD). We characterize parameters essential for sustaining efficient HBD and assess the roles of λRed and the endogenous DNA repair RecA protein in determining the overall efficiency of Pro-AG versus HBD. We also provide proof-of-concept for harnessing HBD as a mitigation tool to delete an engineered genetic cassette should the need arise to do so by precisely excising an inserted cassette to efficiently reverse inactivation of the *bla* target gene function. We show furthermore that the restorative sgRNA mediating HBD can be delivered either by plasmid or engineered phage vehicles. The much expanded range of applications offered by the flexible Pro-MobV system and orthogonal phage delivery should provide a rich tapestry of choices for targeting AR and diverse pathogenicity factors to reduce the virulence of current and newly emerging pathogens, thereby contributing to alleviation of the AR crisis.

## Results

### Consolidating multiple Pro-AG components into a single plasmid

The original Pro-AG system we developed consisted of two low copy number plasmids carrying CRISPR components [1: aTC regulated Cas9 (pCas9); and 2: homology-flanked sgRNA + arabinose-inducible λRed cassettes (pPro-AGAmp)] and a third high copy number target plasmid (pETag)^11^, carrying two AR determinants, the *bla* target locus conferring resistance to Ampicillin (Amp^R^) and a *aacC1* gene cassette conferring resistance to Gentamycin (Gm^R^) (see summary scheme in Supplementary Fig. 1). Inclusion of the non-targeted Gm^R^ marker on the target plasmid permitted selection for gene-edited plasmids in which the *bla* gene was inactivated by insertion of the sgRNA cassette. When bacteria carrying all three plasmids (the two Pro-AG encoding plasmids plus the target plasmid) were grown in the presence of inducers of both Cas9 and the λRed cassette, recovery of Amp^R^ colony-forming units (CFU) was reduced by ~5-logs and all Amp sensitive (Amp^S^) Gm^R^ colonies analyzed carried precise insertions of the sgRNA-encoding cassette (consisting of the sgRNA scaffold and its promoter) in the *bla* target gene.

**Fig. 1:**
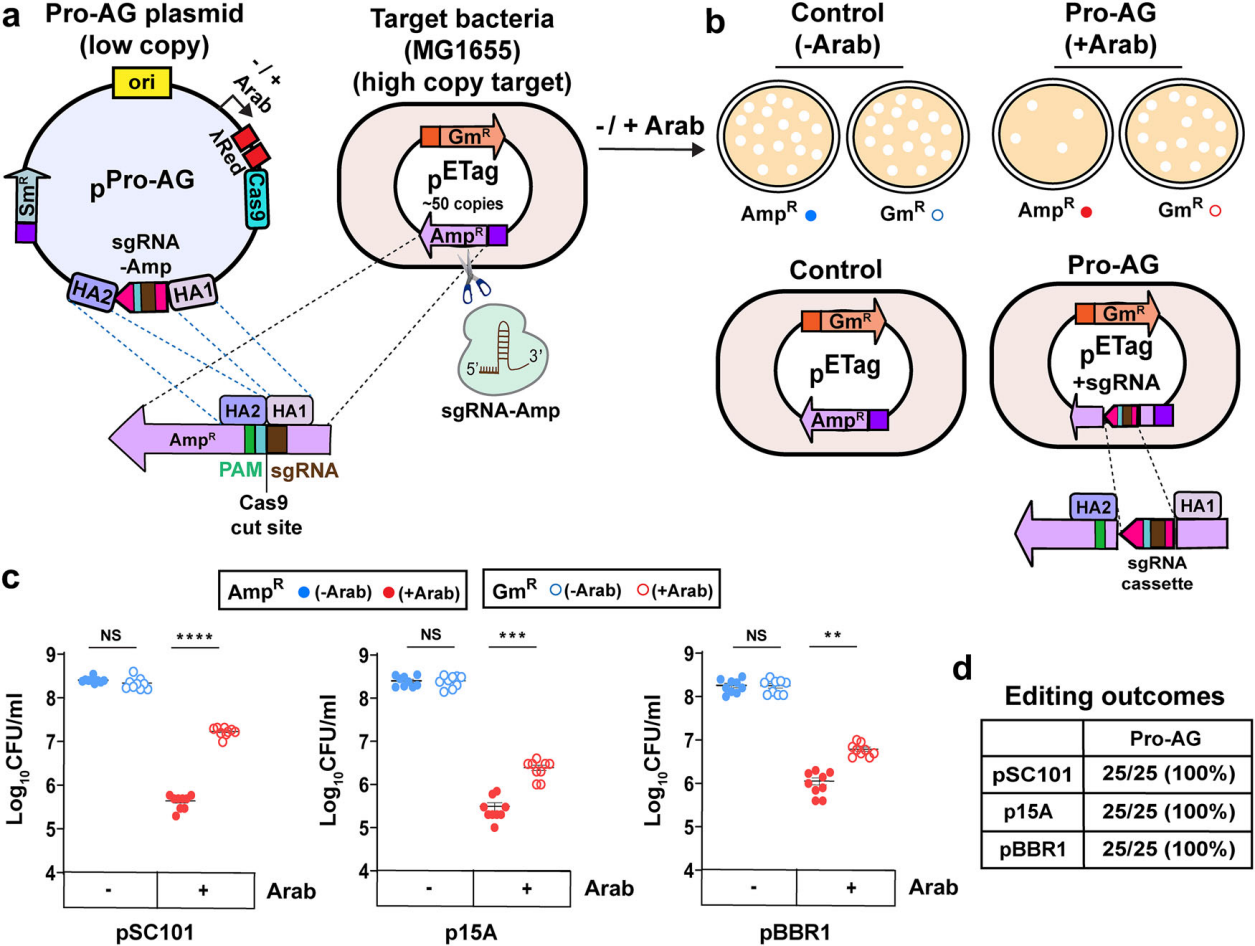
Pro-AG is most effective when launched from a low copy number donor plasmid. **a** Schematic of p^Pro-AG^ and p^ETag^ plasmids. p^Pro-AG^ plasmid maintained under spectinomycin (Sm) selection and carrying arabinose regulated pBAD promoter expressing λRed and Cas9, and constitutive tet promoter to express the sgRNA flanked with the homology arms (HA1 and HA2) targeting the beta-lactamase gene *bla* (Amp^R^) was electroporated into target *E. coli* MG1655 carrying high copy number dual antibiotic-resistant p^ETag^ plasmid (ampicillin, Amp: gentamicin, Gm). The Cas9/sgRNA cleavage site and the protospacer adjacent motif (PAM) are indicated. **b** Scheme of the Control and Pro-AG experiments. Following electroporation, single colonies were grown overnight in the absence (−Arab, open blue dots) or in the presence (+Arab, open red dots) of arabinose for λRed and Cas9 induction. Aliquots were diluted and plated on Amp or Gm plates for colony-forming units (Log_10_CFU/ml) enumeration. In this and most of the following figures, CFU on Amp plates following Control and Pro-AG experiments are represented as solid blue dots and solid red dots while CFU on Gm plates following Control and Pro-AG experiments are represented as open blue dots and open red dots. Also indicated are the schematic of target plasmids in the absence (p^ETag^) and presence (p^ETag+sgRNA^) of Pro-AG events respectively. **c** Recovery of Amp^R^ and Gm^R^ CFU following Control vs Pro-AG when the donor plasmid vegetative origin was pSC101 (~1 copy; left graph), p15A (~8 copies; middle graph) and pBBR1 (~12 copies; right graph). **d** DNA sequence analysis of plasmids isolated from single colonies from Gm plates after Pro-AG events. Data were plotted as the mean ± SEM, representing three independent experiments performed in triplicate and analyzed by Student’s *t*-test. NS not significant (*P* > 0.05); **P < 0.01; ***P < 0.001; ****P < 0.0001.

As a first step in creating a unitary conjugal transfer vector (p^Pro-MobV^) capable of disseminating the Pro-AG system throughout the bacterial population, we consolidated all the Pro-AG components into a single plasmid (p^Pro-AG^: Fig. 1a). Assembling this composite plasmid was not as straightforward as anticipated, requiring iterative efforts to optimize the arrangement of the individual Pro-AG components. Briefly, we tested an array of configurations for co-induction of Cas9 and λRed transgenes and constitutive expression of the sgRNA (see Supplementary Information and Supplementary Fig. 2). In general, we found that when two different conditionally activated genes were carried on the same plasmid (p^ProAG-i-iv^), under all the various configurations we tested, that only one could be efficiently induced. Alternatively, it is possible that premature production of Cas9 preceding accumulation of λRed factors would cut and destroy the target plasmid before λRed functions were active to edit the cut plasmids (Supplementary Fig. 2a–f). This difficulty was overcome, however, when Cas9 and λRed were co-induced as part of a single fusion operon under control of the arabinose-inducible pBAD promoter, wherein Cas9 coding sequences were placed downstream of the λRed operon. In the resulting p^Pro-AG^ plasmid, λRed and Cas9 could be readily co-expressed upon addition of arabinose, while the homology-flanked sgRNA cassette targeting the *bla* gene was constitutively expressed (under control of the *tet* promoter; which contained two palindromic operator sequences but without TetR sequences mediating aTC inducibility) (Fig. 1a).

Motivated by our prior studies that revealed a key role for self-amplification of the sgRNA cassette in achieving efficient Pro-AG events^11^, we generated a series of Pro-AG plasmids of varying reported copy number (ranging from ~1 to 20 copies per cell) using different origins of replication^25–27^. We experimentally confirmed the copy number of three different Pro-AG plasmids (p^SC101^, ~1 copy/cell; p^15A^, ~8 copies/cell; p^BBR1^, ~12 copies/cell) by quantitative PCR (qPCR) and evaluated the performance of each plasmid by transforming them into MG1655 cells carrying the high copy number p^ETag^ target plasmid, which confers resistance to Amp and Gm. After transformation, individual bacterial colonies were selected on agar plates containing triple antibiotics (Amp + Gm + Sm). These colonies were then grown overnight in Luria broth (LB) cultures either supplemented with arabinose to induce the expression of the λRed-Cas9 fusion operon (Pro-AG condition) or without arabinose (Control). Finally, cultures were plated on agar containing either Amp or Gm to determine colony-forming units (CFU) (Supplementary Fig. 3).

When the consolidated Pro-AG components were delivered from a p^Pro-AG^ plasmid carrying the lowest copy number origin (ori pSC101, ~1 copy), we observed ~1000-fold reduction in CFU on Amp plates (solid red dots) and ~100-fold recovery of these CFU on Gm plates (open red dots) in the presence of arabinose. The ~100-fold recovery on Gm plates (open red dots) is due to the disruption of the *bla* gene via copying of the sgRNA-encoding cassette into the sgRNA target site of the *bla* gene (Pro-AG event), while the ~1000-fold reduction in Amp^R^ CFU (solid red dots) results from the combined effects of plasmid destruction (CRISPR event) and *bla* gene disruption (Pro-AG event) (Fig. 1b lower panel, c, left graph), which is comparable to the performance of the original multi-component system^11^. As expected, based on our prior characterization of the multi-component Pro-AG system^11^, all 25 tested single colonies that grew on Gm plates following arabinose addition failed to grow on Amp plates. DNA sequencing analysis revealed that these Amp^S^ Gm^R^ colonies all carried p^ETag+sgRNA^ plasmids with perfect predicted insertions of the sgRNA-encoding cassette into the sgRNA target site of the *bla* gene (Fig. 1d). We observed a similar ~1000-fold reduction of CFU on Amp plates (solid red dots) when using a p^Pro-AG^ plasmid with an intermediate increase in copy number (ori p15A, ~8 copies); however, the recovery of CFU on Gm plates (open red dots) was only ~10-fold greater (Fig. 1c, middle graph). In the case of the medium copy number ori pBBR1 (~12 copies), the number of Amp^R^ CFU (solid red dots) was reduced more modestly by ~100-fold (Fig. 1c, right graph), while the differential recovery of Gm^R^ colonies (open red dots) was only ~10-fold greater. As expected, all tested candidate Pro-AG single colonies that grew on Gm plates failed to grow on Amp plates. DNA sequencing analysis confirmed that all such recovered Gm^R^ Amp^S^ colonies carried perfect insertions of the sgRNA-encoding cassette into the target site on the *bla* gene (Fig. 1d) (p15A origin: 25 out of 25; pBBR1 origin: 25 out of 25). These comparative experiments reveal that having a large difference in copy number between the p^Pro-AG^ launching plasmid and its high copy number target plasmid results in the maximal Pro-AG effect. Thus, when using a higher copy number launching plasmid, a fraction of the potential amplification has already been achieved at the starting point of the experiment, presumably reducing the observed Pro-AG component. We wondered whether the copy number of the inducible λRed-Cas9 gene cassette might impact bacterial growth dynamics and found that this was not the case (Supplementary Fig. 4). These observations are consistent with our prior analysis using dual Pro-AG plasmids wherein a key element underlying the Pro-AG effect was self-amplification of the sgRNA cassette^11^.

### Construction of the conjugal transfer pPro-MobV plasmid

**Fig. 2:**
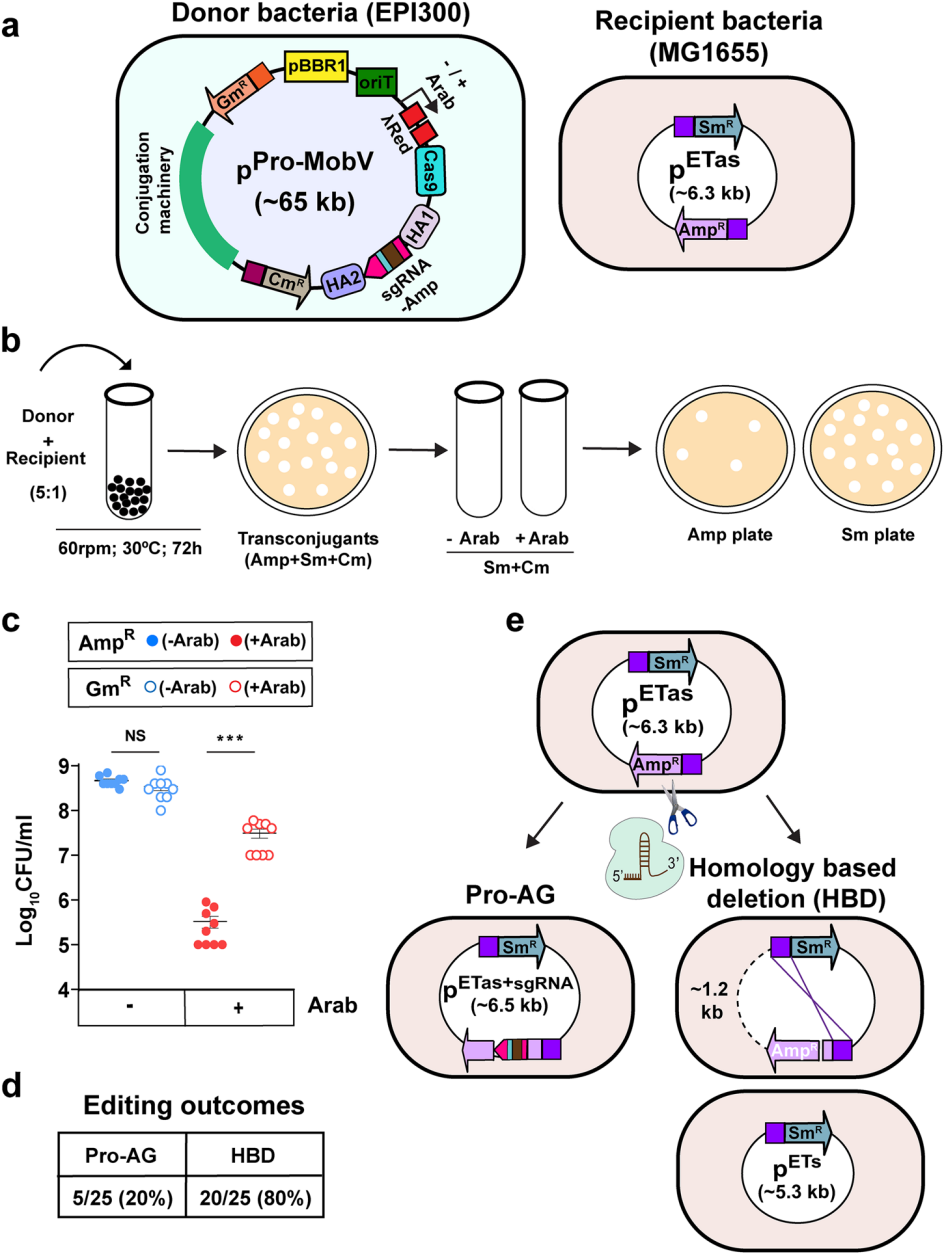
Efficient reduction of Amp^R^ CFU and recovery on Sm plates following transfer of Pro-AG components using conjugal transfer machinery. **a** Schematic of p^Pro-MobV^ and p^ETas^ plasmids. Left, donor *E. coli* EPI300 containing p^Pro-MobV^ includes the following components: medium copy *ori* pBBR1, conjugative origin of transfer (*oriT)*, arabinose-inducible λRed and Cas9, sgRNA targeting *bla* (Amp^R^) flanked with HAs, chloramphenicol resistance (Cm^R^) and Gm^R^ resistance genes. Conjugative machinery, genes required for conjugation are derived from the IncP RK2 conjugative system. Right, *E. coli* MG1655 recipient cells carrying p^ETas^ plasmid. **b** Scheme of conjugation using donor and recipient cells, selection of transconjugants on triple antibiotics plate (Amp + Sm + Cm), overnight culture with and without arabinose and plating on Amp or Sm plates. **c** Recovery of CFU on Amp plates (solid blue and red dots) and Sm plates (open blue and red dots) following Control and Pro-AG experiments. **d** DNA sequence analysis of plasmids isolated from single colonies from Sm plates after Pro-AG events. **e** Scheme depicting HBD-mediated deletion of *bla* locus via recombination between the directly repeated promoters driving expression of the Amp^R^ and Sm^R^ genes. Data were plotted as the mean ± SEM, representing three independent experiments performed in triplicate and analyzed by Student’s *t-* test. NS not significant (*P* > 0.05); ***P < 0.001.

Having consolidated all the Pro-AG components into a single plasmid as described above, we next merged them with the cis-acting conjugal transfer machinery derived from the broad host-range self-transmissible IncP RK2 plasmid^18,23,24^. We constructed the large (~65 kb) p^Pro-MobV^ plasmid consisting of the arabinose-inducible Pro-AG components (λRed-Cas9), a constitutive homology-flanked sgRNA cassette driven by tet promoter, and, a broad host-range origin of replication, genes required for the plasmid maintenance, stability and for conjugation derived from the IncP RK2 conjugative system (referred collectively as conjugation machinery in our study), and a cis-acting *ori*T sequence required for conjugal transfer of the donor plasmid to recipient cells. This donor plasmid also includes two AR markers conferring resistance to Gm and chloramphenicol (Cm) (Fig. 2a left). We chose the pBBR1 as oriV for inclusion in p^Pro-MobV^ even though this origin sustains somewhat lower levels of Pro-AG than plasmids with lower copy number origins since it met the important following criteria: (i) pBBR1 ori does not belong to the incompatibility groups of those plasmids that are heavily used in conjugal gene transfer experiments in bacteria, (ii) it stably maintains the large regions from RK2 necessary to ensure intact Tra functions, and (iii) the pBBR1 replicon has a broad host range sustaining transfer and replication in a wide range of bacterial species^23^.

We verified the predicted overall organization of the p^Pro-MobV^ plasmid by restriction enzyme analysis and then sequenced the entire ~65 kb plasmid to confirm its detailed structure (see Supplementary Fig. 5 for details). We again tested whether bacteria carrying the large p^Pro-MobV^ plasmid grew similarly to those carrying the same Pro-AG components in the smaller p^Pro-AG^ plasmid backbone and observed no significant difference in their growth kinetics (Supplementary Fig. 6).

We next used *E. coli* Epi300 carrying the p^Pro-MobV^ plasmid as the donor bacterial strain and *E. coli* MG1655 carrying the high copy number dual AR bearing Amp^R^ Sm^R^ target plasmid (p^ETas^) as the recipient bacterial strain and performed liquid conjugation assay with beads^18^ (Fig. 2b). We observed ~40% conjugation frequency after mixing donor and recipient cells at a 5:1 ratio and incubating them for 72 h with 60 rpm agitation at 30 °C.

### Conjugal transfer of a Pro-AG cassette efficiently reduces AR in recipient cells by two distinct reinforcing mechanisms

Following conjugation, transconjugants carrying both the p^Pro-MobV^ plasmid and the p^ETas^ target plasmid were selected overnight on low-salt LB (LSLB) agar plates (low salt being optimal for conjugation experiments) with triple antibiotics (Amp+Sm+Cm). Single colonies were regrown in LSLB media either in the absence (Control) or presence of arabinose (ProAG) to induce expression of the λRed-Cas9 fusion operon, and were then plated on either Amp or Sm containing agar plates for CFU enumeration. We observed a ~ 1000-fold reduction of CFU on Amp plates (solid red dots) and ~100-fold recovery on Sm plates (open red dots) (Fig. 2c), which we note is approximately tenfold more efficient than what we had observed in the case of the smaller consolidated intermediate p^Pro-AG^ plasmid carrying the same pBBR1 origin of replication (Fig. 1c, right graph with pBBR1 ori). We expected that all single colonies resulting from induction of the Pro-AG system with arabinose growing on Sm plates, but not on Amp plates, would have undergone precise editing events with the sgRNA cassette inserted precisely into its target site in the *bla* gene. Surprisingly, however, DNA sequence analysis of Sm^R^ Amp^S^ colonies revealed that only ~20% of these colonies displayed typical precise sgRNA insertional events to disrupt *bla* gene function. The remainder of the examined Sm^R^ Amp^S^ colonies (~80%) instead carried a ~1.2 kb deletion of pETas plasmid sequences that removed the entire coding region of the *bla* gene (Fig. 2d). Further analysis of these deletion clones revealed that they had all undergone a precise recombination event between the two directly repeated promoters used to drive expression of the Amp and Sm resistance marker genes, which were nearly identical sequences. We hypothesized that these deletion events were likely the result of Cas9-mediated cleavage in the targeted coding region of the *bla* gene followed by recombination between the two direct repeats of the two AR gene promoters as has been previously observed in the case of low frequency Cas9-induced genomic deletions^28^. We termed this putative recombinogenic event as homology-based deletion (HBD) (Fig. 2e, lower panel). Further complete DNA sequence analysis of pETas plasmids with HBD events revealed minor sequence differences between the Amp and Sm promoters. Thus, the promoter used to drive Sm^R^ differed by three nucleotides after the first 75 nucleotides, resulting in two contiguous direct repeats of ~75 and ~17 nucleotides in the promoter region. The homologous recombination events we analyzed presumably had taken place between either the first ~75 homologous nucleotides or between the second ~17 homologous nucleotides thereby resulting in a stereotyped deletion of ~1.2 kb of intervening DNA sequences between the two promoters. As a result of such recombination events, the *bla* gene was deleted and only the Sm^R^ conferring gene was retained in these smaller plasmids (p^ETs^) (Supplementary Fig. 7 and Fig. 2e, lower panel).

### HBD is independent of bacterial RecA activity, requires Cas9 cleavage between direct repeats, and is enhanced by λRed

**Fig. 3:**
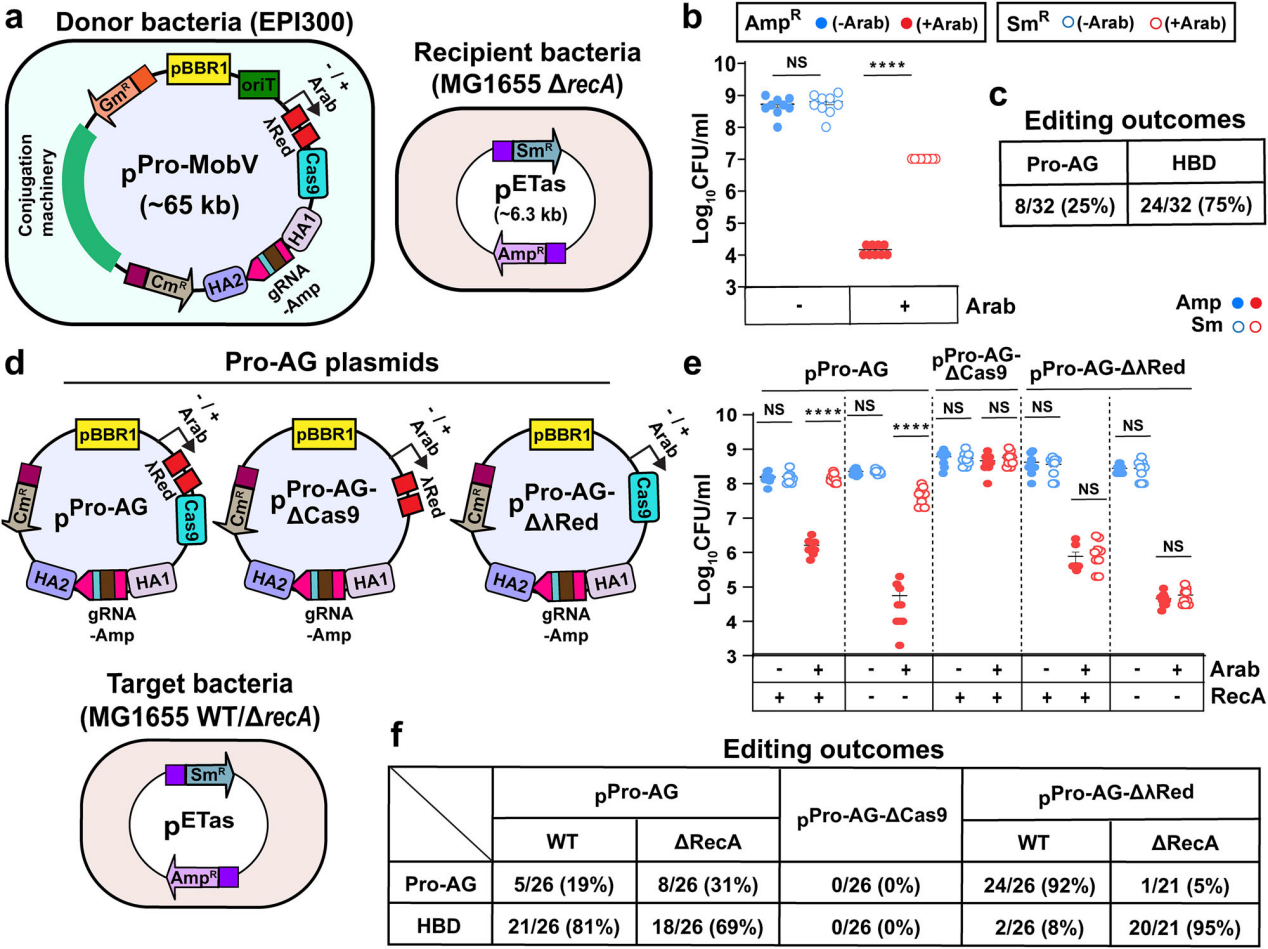
The HBD is sgRNA/Cas9-mediated cleavage dependent and is independent of RecA activity. **a** Donor bacteria EPI300 containing p^Pro-MobV^ constitutes medium copy *ori* pBBR1, conjugative origin of transfer (*oriT)*, arabinose-inducible λRed and Cas9, sgRNA targeting *bla* (Amp^R^) flanked with HAs, chloramphenicol (Cm^R^) and gentamicin (Gm^R^) resistance gene. Conjugative machinery, genes required for conjugation and *E. coli* MG1655 Δ*recA* recipient cells carrying p^ETas^ plasmid. **b** Tabulation of editing outcomes of CFU recovered on Amp plates and Sm plates following Control and Pro-AG experiments. **c** DNA sequence analysis of plasmids isolated from single colonies from Sm plates after Pro-AG events. **d** Schematic of plasmids used to compare HBD events, p^Pro-AG^, p^Pro-AG-ΔCas9^ (Cas9 deleted version of p^Pro-AG^), p^Pro-AG-ΔλRed^ (λRed deleted version of p^Pro-AG^), and target *E. coli* MG1655 WT or isogenic Δ*recA* cells carrying p^ETas^ plasmid. **e** Comparison of CFU following Control and Pro-AG using p^Pro-AG^, p^Pro-AG-ΔCas9^ and p^Pro-AG-ΔλRed^ donor plasmids in WT or Δ*recA* receiver cells. **f** DNA sequence analysis of target plasmids isolated from single colonies from Sm plates after Pro-AG events. Data were plotted as the mean ± SEM, representing three independent experiments performed in triplicate and analyzed by Student’s *t*-test. NS not significant (*P* > 0.05); ****P < 0.0001.

In our previous study^11^ we observed that the endogenous RecA (bacterial DNA repair system) opposed Pro-AG activity. We therefore investigated whether RecA might play a similar role in restricting either Pro-AG or HBD events in our current study by performing a conjugation experiment employing *recA* deficient recipient cells. When Pro-AG components were delivered from a donor bacterial strain carrying the p^Pro-MobV^ conjugal plasmid to p^ETas^; MG1655 Δ*recA* recipient cells (Fig. 3a), we observed ~100,000-fold reduction of Amp^R^ CFU (solid red dots) and a ~1000-fold recovery of Sm^R^ CFU (open red dots) following induction of the Pro-AG system (Fig. 3b). Thus, in the absence of RecA, Pro-AG is enhanced by ~100-fold relative to WT (RecA+) recipient cells (Fig. 2c). DNA sequence analysis of the single Sm^R^ Amp^S^ colonies recovered following arabinose addition revealed 25% had precise sgRNA insertional events while 75% had undergone HBD events (Fig. 3c). We conclude the p^Pro-MobV^ plasmid can effectively target a high copy number antibiotic locus via conjugation and that both Pro-AG and HBD mechanisms are significantly augmented in strains lacking RecA function.

The hypothesized HBD-mediated recombination outcomes observed above led us to investigate whether this efficient phenomenon, like Pro-AG, was dependent on the activities of Cas9 or λRed to carry out recombination between the directly repeated promoter sequences. We analyzed the potential requirements for Cas9 or λRed in the context of the simpler and smaller p^Pro-AG^ plasmid backbone. We constructed two variant plasmids (i) p^Pro-AG-ΔCas9^, in which Cas9 was deleted, and (ii) p^Pro-AG-ΔλRed^, in which the λRed cassette was deleted (Fig. 3d). We transformed bacteria carrying pETas target plasmid with a control (p^Pro-AG^) and the two p^Pro-AG^ variant plasmids, and then assayed recovery of AR colonies on Amp or Sm plates in the presence or absence of arabinose and analyzed target plasmids recovered from Sm^R^ Amp^S^ colonies by DNA sequencing. As expected, the control plasmid generated a similar Pro-AG activity as its closely related counterpart tested in Fig. 1c (pBBR1 ori, right graph), ~100-fold in the presence of arabinose (solid red dots) (Fig. 3e) but, in this case, the molecular outcomes consisted of a mixture of Pro-AG (19%) and HBD (81%) events (Fig. 3f). When Δ*recA* recipient cells were used we again observed a greater reduction in Amp^R^ CFU with arabinose addition (solid red dots) (Fig. 3e), paralleling findings in our previous study and experiments above using a p^Pro-MobV^ multi-component system and the relative proportion of Pro-AG versus HBD was 31 versus 69% (Fig. 3f). One difference in these experiments in which both Pro-AG and HBD can occur relative to those in Fig. 1, where only Pro-AG outcomes can occur, is a notable recovery of Sm^R^ CFU following arabinose addition. This effect may result from nearly all the cut plasmids being repaired either by Pro-AG or HBD. In the absence of RecA, however, some plasmid destruction (greater reduction in Amp^R^ CFU in presence of arabinose, solid red dots) is again observed, which presumably reflects a modest baseline role for this repair pathway in rescuing cleaved plasmid templates.

In the test plasmid lacking Cas9 (p^Pro-AG-ΔCas9^), as expected, we observed no significant reduction of CFU on either Amp (solid red dots) or Sm (open red dots) plates in response to arabinose addition relative to controls (no arabinose, blue dots) (Fig. 3e). All target plasmids isolated from single colonies growing on Sm plates following arabinose addition also grew on Amp plates and DNA sequencing analysis verified that they all carried intact *bla* coding sequences (Fig. 3f). The lack of any type of DNA lesion in the target plasmid in the absence of the Cas9 transgene confirmed that generation of a Cas9-mediated DSB in the target plasmid is indeed required for efficient Pro-AG or HBD.

In contrast to the strict requirement for Cas9, when only the λRed cassette was deleted from the donor plasmid (p^Pro-AG-ΔλRed^), we still observed a ~1000-fold reduction in Amp^R^ CFU (solid red dots), but very little recovery on Sm plates (open red dots) upon arabinose addition (Fig. 3e), suggesting that Cas9 cleavage in the absence of λRed-mediated DNA repair was acting primarily to cut and destroy the target plasmid (similar to results from prior CRISPR-only controls using the multi-component Pro-AG system^11^). Nonetheless, following arabinose addition when a diluted bacterial culture (10^−3^–10^−4^ dilution) was plated on Sm plates, we were able to isolate a minority of colonies that were Sm^R^ and Amp^S^ (36% of Sm^R^ colonies tested). Sequence analysis of those Sm^R^ Amp^S^ plasmids revealed that ~92% of colonies had undergone precise Pro-AG events while the remaining fraction (~8%) had undergone HBD recombinational events (Fig. 3f). We also examined the combined effect of eliminating λRed and endogenous RecA function (Fig. 3e), upon plating arabinose treated diluted bacterial culture (10^−2^–10^−3^ dilution) on Sm plates we again were able to isolate colonies that were Sm^R^ and Amp^S^ (65% of Sm^R^ colonies tested). In this case, we found that 95% of target plasmids had undergone HBD whereas 5% had precise Pro-AG insertions (Fig. 3e, f). The nature of the bacterial DNA repair systems that sustain low levels of Pro-AG and relatively higher rates of HBD in the absence of both λRed and endogenous RecA activity, remain to be determined (see Discussion).

Finally, we also investigated whether the region of promoter homology in the target plasmid was required for HBD and if RecA was still opposing Pro-AG only roles as in the case of HBD events observed above.

Using the same p^Pro-AG^ plasmid but a dual AR target plasmid that lacked direct promoter repeats, p^ETag^, we again observed greater decrement of Amp^R^ CFU in RecA- background with 100% Pro-AG upon arabinose induction (see Supplementary Information and Supplementary Fig. 8).

### HBD-mediated precise deletion of a Pro-AG cassette restores *bla* function

**Fig. 4:**
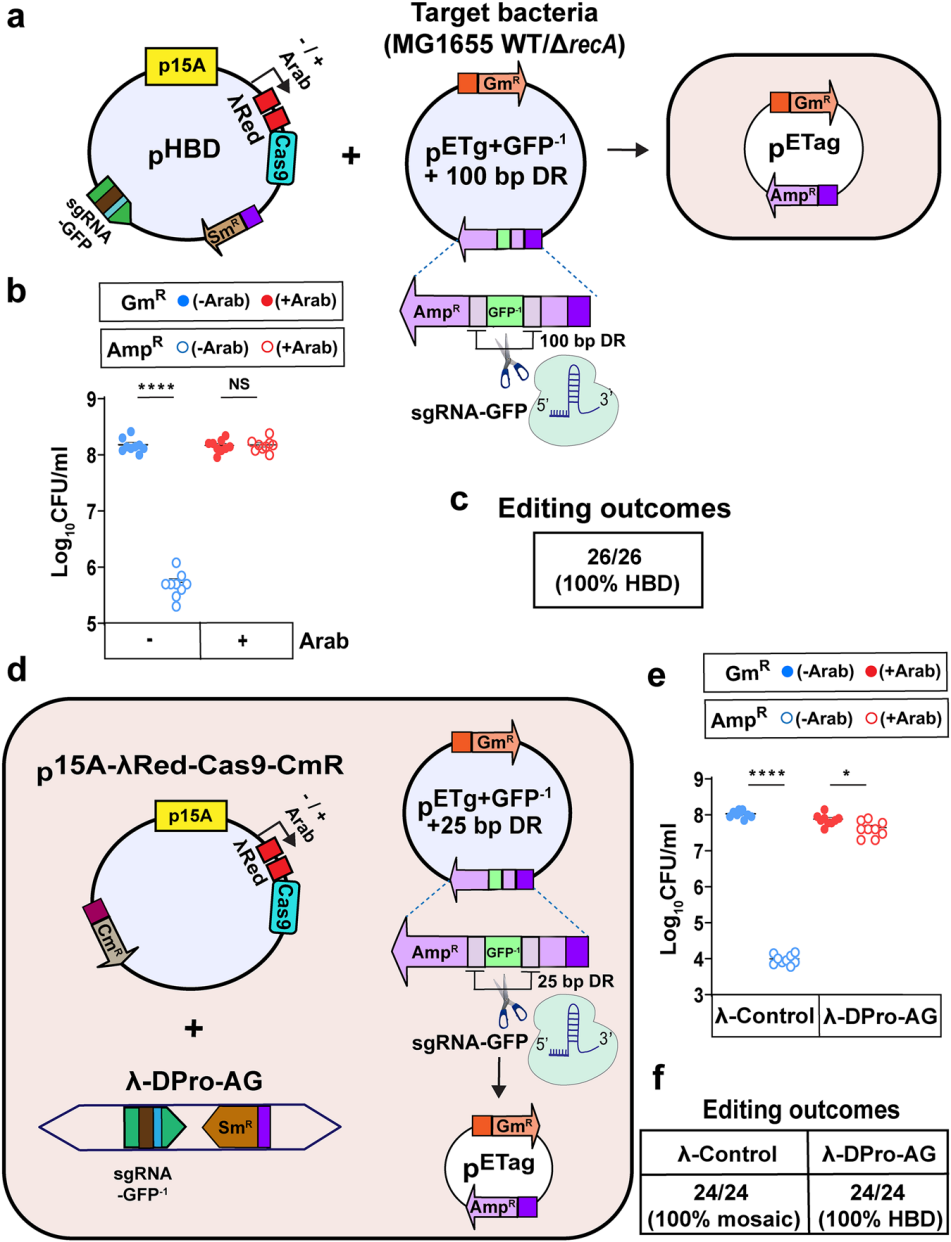
Efficient recovery of Amp resistance using phage delivery system. **a** Schematic of p^HBD^ plasmid carrying p15A *ori*, arabinose-inducible λRed and Cas9, sgRNA-GFP targeting *gfp* and spectinomycin resistance gene (Sm^R^), p^ETg+GFP-1+100bpDR^ and p^ETag^ plasmid (right). **b** Comparison of CFU in the presence or absence of arabinose on Amp or Gm plates. **c** DNA sequence analysis of target plasmids isolated from single colonies from Amp plates in the presence of arabinose. **d** Schematic of p^15A-λRed-Cas9-CmR^ plasmid and λ-DPro-AG phage (left), and p^ETg+GFP-1+25bpDR^ with p^ETag^ plasmid (right). The λ-Control phage carries a nontargeting sgRNA (i.e., which matches no sequences present in either the target plasmid or bacterial genome). **e** Comparison of CFU in the presence of arabinose on Amp or Gm plates with or without the phage carrying sgRNA-GFP. **f** DNA sequence analysis of target plasmids isolated from single colonies from Amp plates. Data were plotted as the mean ± SEM, representing three independent experiments performed in triplicate and analyzed by Student’s *t*-test. NS not significant (*P* > 0.05); *P < 0.01; ****P < 0.0001.

A significant general concern with active genetic systems, such as gene-drives, is mitigation of potentially unintended outcomes. A variety of drive reversal systems have been developed to help address such possibilities^29–31^ including ERACR elements^31^ that delete and replace the drive element with a non-driving cassette, or placing the drive element between directly repeated sequences permitting conditional deletion of the cassette upon nuclease cleavage between them by the eukaryotic Single-Strand Annealing (SSA) pathway^32,33^. Since similar safety concerns could be envisioned for Pro-AG systems in bacteria, we wondered whether it might be possible to harness HBD to cleanly delete a gene cassette inserted into a target site. We thus designed a target plasmid (p^ETg+GFP-1+100bpDR^) with a gene cassette carrying an inactive GFP marker (GFP is toxic when present in high copy number^11^) inserted into the *bla* gene flanked by a 100 bp direct adjacent *bla* sequences (100bpDR) (Fig. 4a; see Supplementary Fig. 9 for detailed information**)**. We also constructed an accompanying mitigation plasmid (p^HBD^) (Fig. 4a) that carries an arabinose-inducible λRed-Cas9 cassette and sgRNA2-GFP (this sgRNA was the most effective of two tested for cleavage of the *gfp* transgene (Supplementary Fig. 10). We reasoned that upon arabinose addition Cas9/sgRNA-GFP would cut the *gfp* target gene resulting in precise recombination between the two direct *bla* repeated sequences, leading to deletion of the intervening *gfp* cassette and restoration of *bla* activity (i.e., Amp^R^).

We established an *E. coli* MG1655 strain carrying the p^ETg+GFP-1+100bpDR^ target plasmid and transformed these bacteria with the p^HBD^ plasmid. When the cells transformed with p^HBD^ plasmid were grown in the presence of arabinose, equivalent numbers of Gm^R^ (solid red dots) and Amp^R^ (open red dots) CFU were recovered indicating that HBD was highly efficient, restoring nearly 100% function of the *bla* target gene (Fig. 4b). Indeed, 100% (26/26) of single colonies isolated from Amp plates in the presence of arabinose had undergone precise HBD events (Fig. 4c).

We also observed recovery of a low level of Amp^R^ CFU (open blue dots) relative to Gm^R^ CFU (solid blue dots) in the absence of arabinose (Fig. 4b). This low level recovery in the absence of arabinose could be largely attributed to leaky expression of the Cas9 + λRed gene cassette. We examined the requirements for Cas9 and λRed by deleting either or both of these components from the p^HBD^ plasmid and found that both genes were required for efficient recovery of Amp^R^ CFU (Supplementary Fig. 11). We still observed basal recovery of Amp^R^ CFU in the absence of Cas9 and λRed (Supplementary Fig. 11) and this basal recovery dropped (by more than a log) when the direct repeat length in the target plasmid was shortened from 100 to 25 bp (see accompanying Supplemental Information and Supplementary Fig. 12). Recovery of Amp^R^ CFU (open blue dots) in the absence of arabinose with p^HBD^ (Fig. 4b) initially suggested that this low level of recovery could be largely attributed to leaky expression of the Cas9+λRed gene cassette. However, because we still observed basal Amp^R^ CFU even in the absence of Cas9+λRed, and because such recovery decreased when the direct repeat length in the target plasmid was decreased, we hypothesize that such Cas9 and λRed independent recovery of basal Amp^R^ CFU could be attributed to slipped misalignment during DNA replication^34^ as well as leaky expression of the Cas9+λRed gene cassette when that cassette is present. Furthermore, deep analysis of the sequencing results of the target plasmids isolated from single colonies on the Amp plates in the absence of arabinose addition confirmed that when Cas9 was included in the plasmids (p^HBD^ and p^HBD-ΔλRed^) 100% of colonies had undergone complete HBD whereas ~34% were HBD events (intact *bla* sequence on the target plasmid) and ~66% were unedited plasmids (intact GFP sequence on the target plasmid) for the p^HBD-ΔCas9^ plasmid and ~28% were HBD events and ~72% were unedited plasmids for the p^HBD-ΔCas9-ΔλRed^ plasmid (see Supplementary Fig. 13 and Supplemental Information for a more detailed explanation) resulting in mosaic colonies whenever Cas9 was absent in the p^HBD^ plasmids. These results suggest the involvement of leaky expression of Cas9 and slipped misalignment during DNA replication resulting in 100% HBD events in Cas9 carrying plasmids and only ~28% - ~34% HBD events, possibly by the slipped misalignment during DNA replication in the absence of Cas9.

### Phage delivery of the HBD inducing sgRNA can also restore AR

In addition to conjugal transfer systems such as the p^Pro-MobV^ plasmid system, bacteriophages (or phage) offer an orthogonal means for delivering CRISPR components to bacteria^35–37^. We hypothesized that we could exploit phage to create a dual carriage system to mediate HBD in the context of the AR-restoration assay described above. We tested this possibility by replacing nonessential sequences from phage λ with the same sgRNA2-GFP cassette carried in the p^HBD^ plasmid and Sm^R^ (λ-DPro-AG) or a control nontargeting sgRNA and Sm^R^ (λ-Control) (Supplementary Fig. 14). We infected bacteria carrying the p^ETg+GFP-1+25bpDR^ target and p^15A-λRed-Cas9^ plasmids with these lysogenic phages, and assayed resulting lysogens for restoration of Amp^R^ (Fig. 4d). We found that such phage delivery of sgRNA-GFP significantly reverted Amp sensitivity back to Amp resistance (open red dots) (Fig. 4e) and that all such tested colonies displayed a precise deletion of the *gfp* target cassette (100%: 24/24) (Fig. 4f), indicative of Cas9/sgRNA-mediated cleavage of the *gfp* target site. Consistent with our prior experiments using the plasmid-based p^HBD^ system, when the target cells were infected with control phage carrying the nontargeting sgRNA, we only recovered a low baseline level of mosaic colonies (100%: 24/24) that again may have resulted from slipped misalignment during DNA replication (Fig. 4f, see Discussion).

## Discussion

In this study, we develop and extensively characterize a flexible multi-functional Pro-AG platform (p^Pro-MobV^) for disseminating a self-amplifying genetic system through bacterial population to reduce the prevalence of antibiotic resistance. The p^Pro-MobV^ conjugal plasmid consolidates the key Pro-AG components (an inducible λRed-Cas9 cassette and constitutive homology-flanked sgRNA) with conjugal transfer machinery into a single mobile genetic element. We demonstrate that the intermediate copy number broad host-range p^Pro-MobV^ plasmid can be transferred from donor cells into recipient cells carrying a high copy number plasmid to efficiently inactivate a *bla* target gene conferring resistance to ampicillin. We also demonstrate the potential for dual carriage of CRISPR components by either plasmid or phage vehicles in a novel highly sensitive AR-restoration assay. These and related systems offer a broad range of future potential applications for modifying bacterial genotypes at a population level.

In contrast to prior CRISPR approaches that either target chromosomal virulence genes or plasmid-encoded AR genes for destruction^38,39^, Pro-AG disrupts the AR genes by precise insertion of a sgRNA cassette into the targeted cleavage site leaving the target plasmid (or chromosome) intact. There are two basic advantages of the Pro-AG system in leaving the target plasmid intact. First, as we have shown previously, the efficiency of insertionally inactivating an AR gene is 2–3 logs more efficient than destroying the entire plasmid. Second, many conjugal plasmids carry protective toxin-anti-toxin systems that would kill bacteria failing to transmit them to daughter cells which would further limit the efficiency of the cut and destroy strategy. Leaving the target plasmid intact while eliminating its most undesired cargo provides an alternative approach that would avoid these challenges. It might also be possible, however, to devise a method to supply the anti-toxin without the toxin, which should help the cut-and destroy approach. Since many large conjugal transfer plasmids are low copy number, this may also work in favor of improving the efficiency of the cut-and-destroy approach relative to using it to eliminate high copy number targets.

In this study, we also elucidate a highly efficient activity of p^Pro-MobV^ (or p^Pro-AG^) acting in parallel to Pro-AG, referred to as HBD, that promotes homologous recombination between directly repeated sequences and can be employed in combination with Pro-AG to expand the range of editing performed by this system. We determined that HBD depends on Cas9-mediated target site cleavage, requires direct repeats of ~25–100 bp of homologous sequences for recombination, and is greatly stimulated by λRed-mediated repair of DSBs. We also provide proof-of concept for harnessing HBD in a mitigating mode to cleanly delete a target gene cassette resulting in highly efficient (~100%) restoration of *bla* activity. HBD could also be harnessed to delete a variety of duplicated genetic elements that contribute to AR^40^ including genes encoding AR target proteins^41^ or efflux pumps that eject the antibiotic from bacterial cells^42^ as well as AR genes carried on mobile genomic elements that can duplicate themselves^43^.

Our analysis also revealed a hierarchy of DNA repair pathways relevant to both Pro-AG and HBD.

Thus, the activation of λRed was required for both efficient Pro-AG and HBD events and these activities were opposed by the endogenous bacterial RecA pathway, consistent with prior observation using the multi-component Pro-AG system^11^. HBD events were favored over Pro-AG when the target plasmid carried a direct repeat and the HBD was not dependent on the endogenous bacterial RecA pathway. While the absence of λRed, but presence of endogenous RecA, favored rare events resulting in relatively higher Pro-AG over HBD, the reverse was observed in the absence of both systems. The basis for these rare events and alternative outcome biases is worth further investigation. The very low level of HBD events in the absence of both λRed and endogenous RecA was also revealed in the highly sensitive assay system wherein HBD was employed to restore Amp^R^. The nature of this basal repair pathway merits further investigation and may reflect a previously described mechanism involving slipped misalignment during DNA replication^34^. One line of evidence supporting this hypothesis is that levels of basal HBD detected in the assay were reduced when using shorter flanking repeat sequences, a phenomenon also observed with replication slippage. The SbcBCD complex is another repair pathway that may warrant further examination, particularly given that this system has been implicated in resolution of DNA hairpins during replication created by palindromic sequences^44,45^.

One potential concern regarding the Pro-MobV system is how stable it might be or whether the Pro-AG components or conjugal transfer machinery might carry fitness costs limiting its spread. We did not observe any significant differences in the growth of bacteria carrying either the λRed-Cas9 inducible cassette on plasmids with differing copy number, nor between the large p^Pro-MobV^ plasmid and its smaller non-conjugating p^Pro-AG^ counterpart, although subtle fitness differences could be more sensitively assessed in the future by direct competition experiments between bacterial strains grown together in the same flask. We also note that CRISPR systems are carried on many naturally occurring plasmids^46^. With regard to the issue of Pro-AG stability, in a previous study^11^ we assessed the basis for rare escaper colonies in which the Pro-AG system failed which revealed that approximately half of such mutations disrupted the sgRNA cassette and that the others likely were due to inactivation of the Cas9 cassette. We expect that similar types of mutations could arise in the p^Pro-MobV^ plasmid as well as those disabling either trans or cis-acting conjugal transfer machinery. Future refinements of the Pro-MobV platform could include dual carriage of critical Pro-AG components such as Cas9, sgRNAs, λRed or conjugal transfer components such as distinct oriT sequence that could be placed either within the plasmid itself or in a separate parallel acting element including an orthogonal phage. Inclusion of endogenous CRISPR promoters, toxin-anti-toxin systems and additional plasmid partitioning systems could also aid in stabilizing inheritance of such systems. As conjugation frequencies in our current study using Epi donor and MG1655 as receiver strains over the limited time course of the experiments were less than complete (~40%), it did nonetheless reduce the prevalence of antibiotic resistance by three to five logs depending on the genotype of the receiver bacterial strains. Further optimization of this system, which in principle should be achievable^18^ should enable use of our new HGT-Pro-AG system to be employed for future practical applications.

Widespread use of antibiotics in contexts such as animal husbandry or maintenance of inland fish farms can lead to environmental consequences^47–49^ and transfer of AR genes to humans^48,50,51^. The consolidated unitary conjugal Pro-AG platform described in this study could be efficiently used to target pathogenic bacteria carrying antibiotic resistance or any other virulence factor to reduce the prevalence of AR or pathogenicity in a sequence-specific fashion in these various contexts. Our strategy offers several potential advantages over broad spectrum traditional antibiotics that kill both pathogenic and commensal bacteria indiscriminately, and which can indirectly accelerate the growth of resistant bacteria and alter the microbiome^52,53^. Owing to the simplicity, specificity, and versatility of our flexible and efficient active genetic toolkit, the Pro-MobV platform could be adapted for diverse applications such as scrubbing antibiotic resistance genes while at the same time deleting pathogenicity islands, AR associated duplications of efflux pumps, or performing subtle edits to revert evolving virulence phenotypes in bacterial populations. HBD using engineered phage delivery systems offers an orthogonal means for remediation by cleanly deleting an undesired gene cassette from either a plasmid or potentially a bacterial genome. The future integration of both conjugal and phage delivery systems offers various opportunities including robust parallel delivery of key CRISPR components and opportunities to evolve yet more efficient dual carriage systems. Future applications of this Swiss Army knife like technology include measures to reduce AR in the environment and potentially in clinical settings for targeting AR and other virulence factors in bacteria causing intermittent disease, such as tuberculosis or low-level persistent infections, such as chronic urinary track or recurring cryptic epidermal infections.

## Methods

### Strains and culture conditions

*E. coli* strain MG1655 WT and *ΔrecA* were provided by Dr. B. Palsson and Dr. Susan Lovett Laboratories, respectively, as mentioned in our previous study^11^ and were grown in LB medium. *E. coli* strain EPI300 harboring pNuc-cis plasmid was obtained from Dr. David R. Edgell^18^ laboratory and was maintained in LSLB medium. Antibiotics were added as follows unless otherwise described in the figure legends: ampicillin (Amp, 100 µg/ml), gentamicin (Gm, 10 µg/ml), chloramphenicol (Cm, 25 µg/ml), and spectinomycin (Sm, 50 µg/ml). If the plasmids carried the pBAD promoter, 0.2% D-glucose (Sigma) was added to the agar plates in all the plating conditions. Similarly, 0.2% D-glucose was added to shut down the pBAD promoter, followed by the addition of 0.2% L-arabinose for induction in all culture conditions. All the experiments were always carried out at 30 °C.

### Plasmid construction and transformation

All plasmids used in this study were generated using either Gibson assembly or restriction enzyme digestion and are listed in Supplementary Table 1. Following Gibson assembly or ligation, all reactions were transformed into NEB Stable Competent *E. coli* (C3040H) and the integrity of constructs verified by restriction enzyme and sequence analysis.

### *E. coli* MG1655 transformation

*E. coli* MG1655 electrocompetent cells were prepared as previously described (Short Protocols in Molecular Biology, Chapter 1). Aliquoted competent cells were gently thawed on ice and 20 ng of each plasmid DNA prepared using QIAprep Spin Miniprep Kit (Qiagen) were added followed by electroporation using 1 mm Gene Pulser cuvette (Bio-Rad) at 1.6 kV and were resuspended in super-optimal broth with catabolite repression media (SOC). Cells were grown for 2 h at 30 °C, and serial dilutions of cells were plated on LB agar plates with the appropriate antibiotic and were then grown at 30 °C for 24 h. For all the p^Pro-AG^ and p^HBD^ plasmids carrying arabinose-inducible λRed-Cas9 cassette, glucose was added to the agar plates along with antibiotics.

### p^Pro-MobV^ plasmid construction

The p^Pro-MobV^ plasmid was constructed by digestion of pNuc-cis plasmid^18^ using AvrII, BstEII and StuI restriction enzymes. The Pro-AG components, arabinose-inducible **λ**Red-Cas9 cassette and constitutive homology-flanked sgRNA for *bla* locus was prepared after digestion of p^Pro-AG^ plasmid using AvrII and BstEII restriction enzymes. The digested vector and insert were ligated using ElectroLigase (NEB). The ligated product was electroporated into TransforMax EPI300 Electrocompetent *E.coli* (Lucigen) as described earlier, and cells were resuspended in super-optimal broth with catabolite repression media. Cells were grown for 2 h at 30 °C, and serial dilutions of cells were plated on LB agar plates containing Cm and Gm and grown at 30 °C for 24 h. Single colony was grown overnight in LSLB containing Cm and Gm and the plasmid was isolated using the QIAprep kit following Sodium acetate and Isopropanol precipitation. Isolated pPro-MobV plasmid was digested with EcoRI, and the expected digestion pattern was analyzed using 0.8% agarose gel. The whole plasmid was then sequence verified by Primordium, and the nucleotide sequence was deposited in GenBank (accession number: PV588693).

### Induction of Cas9 and λRed enzymes in culture conditions

Single *E. coli* colonies were resuspended in liquid media and grown overnight as described previously^11^. When required, 100 ng/ml anhydrotetracycline (Abcam) was added to induce the tet promoter in the broth media during editing steps. If the plasmids carried the pBAD promoter, 0.2% D-glucose was added to shut down the pBAD promoter, followed by the addition of 0.2% L-arabinose for induction to the broth media during editing steps (see Supplementary Fig. 3).

### Plasmid copy number determination

*E. coli* MG1655 strains carrying p^Pro-AG^ plasmids with different replication origins (as shown in Fig. 1) were grown overnight and diluted 1:100 into fresh broth media containing Sm. Cells were harvested at mid-exponential phase (OD₆₀₀ ≈0.5). Total DNA was isolated using the QIAamp DNA Mini Kit (Qiagen) according to the manufacturer’s protocol for bacterial samples. DNA concentration was measured with a NanoDrop spectrophotometer, and 20 ng of total DNA per reaction was used for quantitative PCR (qPCR). Total DNA from plasmid-free MG1655 served as the negative control. Plasmid copy number was quantified by qPCR using two targets: *dxs* (a single-copy chromosomal gene; primers: F–CGAGAAACTGGCGATCCTTA, R–CTTCATCAAGCGGTTTCACA) and *Sm*^*R*^ (a plasmid-encoded marker; primers: F–TGAGGCGCTAAATGAAACCT, R–TACTGCGCTGTACCAAATGC). Mean Cq values from triplicate reactions were obtained for each gene per sample. The difference between chromosomal and plasmid Cq values was calculated as: ΔCq = Cq_dxs − Cq_SmR. Assuming 100% PCR efficiency (i.e., a two-fold amplification per cycle), plasmid copy number per chromosome equivalent was estimated as: copy number = 2^ΔCq. Negative ΔCq values indicate lower abundance of the plasmid marker relative to the reference gene. No amplification of the plasmid-specific SmR target was observed in plasmid-free controls.

### *E. coli* EPI300 to *E. coli* MG1655 conjugation

EPI300 cells containing p^Pro-MobV^ plasmids were used as donor bacteria, and MG1655 cells containing p^ETas^ plasmids were used as receiver cells and grown overnight in LSLB media to saturation. Tubes containing 5 mL LSLB media with glucose and 1 gm glass beads (0.5 mm diameter; Research Products International) were inoculated with 25 µl saturated EPI300 and 5 µl saturated MG1655 (5:1 ratio). Conjugations were carried out for 72 h at 30 °C with 60 rpm without antibiotics. Cultures were homogenized by vortexing, and 10 µl of serially diluted cultures were spotted on Cm plates for donors, on Amp+Sm agar plates for receivers and Amp+Sm+Cm agar plates for transconjugants. Plates were incubated overnight at 30 °C, and colonies were counted manually. Transconjugants were picked from the triple antibiotic plates and resuspended in 60 µl LSLB media, and were grown overnight in 5 ml LSLB cultures containing glucose for control experiments, whereas arabinose was added for Pro-AG experiments (see Fig. 2 for details).

### λ-D-ProAG phage construction and infection

A nonessential portion of λ cI857’s genome was removed between positions 21,766 bp and 26,900 bp to make room for Pro-AG elements. cI857 was chosen because it has a mutation in the *cI* gene that allows heat induction of lytic replication. The deletion was achieved by using two sgRNAs and arabinose-inducible λ-Red and Cas9 system (Supplementary Fig. 14), and either nontargeting sgRNA or GFP-sgRNA and Sm^R^ cassettes were incorporated into the edited phage to generate λ-Control and λ-DPro-AG phages, respectively. The λ-Control and λ-DPro-AG phages were heat-induced and used to infect naïve MG-WT cells to generate stable lysogens for phage production. A second set of MG-WT cells were transformed with p^ETg+GFP-1+25bpDR^ and p^15A-λRed-Cas9^ plasmids (Fig. 4d), and the cells were plated on LB plates containing Gm+Cm and grown overnight. Single colonies were then grown overnight in LB cultures containing Gm+Cm. Bacteria cultures were then infected with phage particles (1.2 × 10^9^ phage particles and 2 × 10^8^ bacterial culture) in Tris-LB+Gm+Cm+Sm+arabinose media for 24 h. The cultures were then plated on Gm or Amp plates and incubated at 30 °C overnight, colonies were counted and single Amp^R^ colonies were isolated and grown overnight in LB+Amp media. Plasmids were isolated from individual colonies and Sanger sequenced.

### *E. coli* colony counts

For CFU determination, 25 µl of overnight culture was serially diluted, and 10 µl was spotted in triplicate on LB plates with appropriate antibiotics as described previously^11^.

### Sequence analysis of Pro-AG and HBD editing events

For Pro-AG and HBD events, p^ETag^, p^ETas^ or p^ETg+GFP-1+100bpDR^ plasmids were extracted using QIAprep kit from single *E. coli* colonies that were either Amp^S^/Gm^R^ or Amp^S^/Sm^R^ after Pro-AG (+arabinose) experiments and were Amp^R^/Gm^R^ after HBD (± arabinose) experiments. The plasmids were either Sanger (Genwiz) sequenced using a specific primer or whole plasmids were sequenced (Primordium) and the editing events were identified.

### Deep sequence analysis of mosaic sequences

For all control (-arabinose) experiments involving p^HBD^ and its variant donor plasmids, target plasmid p^ETg+GFP-1+100bpDR^ was extracted using Monarch plasmid miniprep kit from single *E. coli* colonies that were Amp^R^/Gm^R^. The plasmids were digested using Not1 (NEB) restriction enzyme. Digested plasmids were run on agarose gel, and only ~6.8 kb bands that appeared when p^HBD^ or p^HBD-ΔλRed^ were used as donor plasmids, or ~6.8 and ~6 kb bands that appeared when p^HBD-ΔCas9^ or p^HBD-ΔCas9-ΔλRed^ were used as donor plasmids were extracted using Monarch DNA gel extraction kit (NEB) and sent for sequencing (Plasmidsaurus). The FASTQ sequences for all 4 conditions were aligned with the reference p^ETg+GFP-1+100bpDR^ plasmid sequence, and the ratio of HBD and Mosaic events were analyzed.

#### Statistical analysis and data presentation

GraphPad Prism 10 was used for all statistical analyses and graphical visualization of data. All the graphical presentation of figures was generated using Adobe Illustrator. SnapGene was used to generate a plasmid map if applicable.

## Supplementary information

Supplementary information

Source Data file

## Data Availability

We thank David R. Edgell Laboratory for providing us the pNuc-*cis* conjugation plasmid. These studies were supported by the Tata Institutes for Genetics and Society - University of California San Diego and NIH grants R01GM117321, R01GM144608, R01AI162911 awarded to Ethan Bier, as well as by Howard Hughes Medical Institute Emerging Pathogen Initiative 311169 awarded to Justin Meyer.
