# Supplementary material for: A conjugal gene drive-like system efficiently suppresses antibiotic resistance in a bacterial population
Source: NPJ Antimicrob Resist. 2026 Feb 2;4:8. doi: 10.1038/s44259-026-00181-z (PMC12864801; doi:10.1038/s44259-026-00181-z)
Supplement: Supplementary file 1 — Supplementary information [file 44259_2026_181_MOESM1_ESM.docx]

**Supplementary Information:**

**A conjugal gene drive-like system efficiently suppresses antibiotic resistance in a bacterial population**

Saluja Kaduwal^1,2^, Elizabeth C. Stuart^3^, Ankush Auradkar^1^, Seth Washabaugh^1^, Justin R. Meyer^3^, and Ethan Bier^1,2*^

**Supplementary Text**

In initial attempts to create a unitary pPro-AG vector we simply combined all of the separate components carried on the two original Pro-AG donor plasmids using a variety of promoters and orientations to express the Cas9, homology-flanked sgRNA, and λRed transgenes (**Supplementary Fig. 2a**). Curiously, none of the many tested configurations using aTC regulated Cas9 (tet promoter containing two palindromic operator sequences and tetracycline repressor TetR cassette) and arabinose regulated λRed (pBAD promoter) expression yielded the desired efficient Pro-AG activity (high Gm^R^ and low Amp^R^ colonies) upon stimulation of cells with both aTC (to activate Cas9 expression) and arabinose (to activate expression of the λRed cassette). For example, when p^Pro-AG-i^ low copy number plasmid was transformed into MG1655 cells carrying the high copy number target plasmid (_p_^ETag^), with dual AR markers, which confers resistance to Amp and Gm like in our previous study ^1^ (**Supplementary Fig. 1 and 2a-2c**) we observed a ~100-fold reduction of Amp^R^ CFU after Cas9 induction with aTC (solid purple dots) but when both Cas9 and λRed were induced, we observed a similar reduction of Amp^R^ and Gm^R^ CFU (solid red dots) (**Supplementary Fig. 2c**). When single colonies that grew on Gm plates after induction of Cas9 and λRed were grown on Amp plates all of the single colonies grew on Amp plates and the DNA sequencing analysis of those plasmids showed an intact Amp^R^ coding sequence (**Supplementary Fig. 2f**). Similar results were observed using the p^Pro-AG-ii^ another low copy plasmid and p^Pro-AG-iii^ a medium copy plasmid (**Supplementary Fig. 2a**). Also the single colonies that grew on Gm plates after induction of Cas9 and λRed grew on Amp plates. Next, we made a p^Pro-AG-Cas9 flipped-iv^ donor plasmid where the orientation of aTC-inducible tet promoter and Cas9 cassette was flipped, we still observed similar results as when we used p^Pro-AG-iii^ as a donor plasmid (**Supplementary Fig. 2a**). Interestingly, when we constructed p^Pro-AG-v^ donor plasmid where we used the arabinose inducible pBAD promoters to induce the expression of Cas9 and λRed we observed a ~100-fold reduction of Amp^R^ CFU (solid red dots) and ~10-fold recovery on Gm plates (open red dots) when both Cas9 and λRed were induced (**Supplementary Fig. 2d, 2e right graph**). All of the single colonies that grew on Gm plates but failed to grow on Amp plates had a precise insertion of sgRNA cassette at its target site in the p^ETag^ plasmid (**Supplementary Fig. 2f**).

The results in **Fig. 3b** and **Fig. 3e** using p^Pro-MobV^ donor plasmid and small Pro-AG WT plasmid in Δ*recA* cells where we observed greater reduction in Amp^R^ CFU compared to WT *recA*+ cells led us to investigate if such decrement in Amp^R^ CFU in the presence of arabinose is precisely because of the homology region in the target plasmid. To investigate this, we performed a similar experiment using a Pro-AG plasmid and a dual AR target plasmid that lacked direct promoter repeats, such as in the case of p^ETag^ (**Supplementary Fig. 8a)**. We observed greater reduction of Amp^R^ CFU (~4-logs) in a Δ*recA* background (solid red dots) than in WT *recA*+ cells (~2-logs) (solid red dots) (**Supplementary Fig. 8b)**. As expected in this simplified configuration, all *recA*^±^ Amp^S^ Gm^R^ colonies analyzed carried precise sgRNA cassette insertions (Pro-AG only events) (**Supplementary Fig. 8c).**

When Cas9 deleted pHBD variant plasmid p^HBD-ΔCas9^ was transformed into MG1655 cells carrying p^ETg+GFP-1+100bpDR^ target plasmid (**Supplementary** **Fig. 11a**), Gm^R^ CFUs were similar for both presence (solid red dots) and absence of arabinose (solid blue dots). On Amp plates, however, ~ 4-logs fewer Amp^R^ CFU were recovered in the absence of arabinose (open blue dots), while in the presence of arabinose, Amp^R^ CFU were reduced by ~ 2-logs compared to Gm^R^ CFU (open red dots) (**Supplementary** **Fig. 11b**). Sanger sequencing analysis of Amp^R^ individual colonies recovered from both the presence (26/26) or absence (10/10) of arabinose revealed a mosaic pattern, consisting of a mixture of unedited (intact GFP sequence) and precisely edited (intact *bla* sequence) plasmids in each colony **(Supplementary** **Fig. 11c)**. We attribute the greater number of Amp^R^ CFU following arabinose addition to cleavage-independent λRed-mediated recombination between the two direct repeats. In the absence of λRed cassette (p^HBD-λRed^) (**Supplementary** **Fig. 11a**), fewer CFU were recovered on either Gm (solid red dots) or Amp plates (open red dots) in presence of arabinose (the effect being greater for Amp^R^ CFU) (**Supplementary** **Fig. 11b**). The reduction of Gm^R^ CFU in presence of arabinose presumably reflects Cas9-mediated plasmid cleavage and destruction. All Amp^R^ colonies recovered had undergone 100 % precise HBD-mediated deletion (10/10 in absence of arabinose and 26/26 in presence of arabinose) **(Supplementary** **Fig. 11c)**. As in the case of deletion of only Cas9, deletion of both Cas9 and λRed cassettes from the p^HBD^ plasmid **(**p^HBD-ΔCas9-ΔλRed^) **(Supplementary** **Fig. 11a)** resulted in ~4-logs reduction of Amp^R^ CFU (open red dots) compared to Gm^R^ CFU (solid blue dots) (**Supplementary** **Fig 11b**). Sequencing of single Amp^R^ colonies revealed a mosaic phenotype in which only a fraction of plasmids in each colony had undergone deletion of the target cassette (100 %: 26/26) **(Supplementary** **Fig. 11c)**. These results suggest that a yet less efficient cleavage (Cas9) independent and λRed independent recombinogenic mechanism acted under these conditions. Hence, we investigated if RecA was involved in HBD-mediated recovery of Amp^R^ CFU upon arabinose addition by repeating the above analysis in *ΔrecA* MG1655 cells. In these experiments, we again observed a basal level of Amp^R^ CFU recovery in the absence of arabinose (open blue dots) and full rescue of *bla* gene activity via precise HBD (100 %: 26/26 colonies) in its presence (open red dots) (**Supplementary** **Fig. 11b and 11c)**) indicating that the above observed HBD restoration of *bla* gene (Amp^R^ colonies) is RecA-independent.

Since we observed that HBD in above experiments were RecA independent we wondered whether basal levels of Amp^R^ CFU recovered in above experiments in the absence of arabinose might be mediated by a process referred to as slipped misalignment during DNA replication ^2^ , which is highly dependent on the length of the repeat and the distance between the repeats ^3, 4^. We tested for such length dependence by reducing the size of the direct repeats from 100 bp to 50 bp and 25 bp and found indeed that the basal recovery of Amp^R^ CFU dropped by more than a log when the direct repeat length was shortened to 25 bp (open blue dots) (**Supplementary Fig. 12a and 12b**), and all the target plasmid sequencing in the presence of arabinose had 100 % (15/15) HBD only events (**Supplementary Fig. 12c**).

In order to deeply analyze the events in the target plasmid in the absence of arabinose, we performed comparative deep analysis of the target plasmid sequence for the p^HBD^, p^HBD-ΔCas9^, p^HBD-ΔλRed^ and p^HBD-ΔCas9-ΔλRed^ donor plasmids (See Materials and Methods section). Analysis of the FASTQ sequencing results confirmed that when Cas9 was present in the plasmids (p^HBD^ and p^HBD-ΔλRed^) 100 % of colonies had undergone complete HBD (100 % of sequences analyzed) whereas only ~34 % and ~28 % HBD events were observed in single colonies when using the Cas9-deleted p^HBD-ΔCas9^ and p^HBD-ΔCas9-ΔλRed^ plasmids **(Supplementary Fig. 13)**. The observed, only ~ 30% HBD events where Cas9 is absent in the plasmids (p^HBD-ΔCas9^ and p^HBD-ΔCas9-ΔλRed^), suggest that this inefficient cleavage-independent process results in mosaic colonies with only a fraction of the plasmids having undergone HBD presumably by slipped misalignment during DNA replication in the absence of λRed and by both - the slipped misalignment during DNA replication and λRed whenever λRed is present. The observed 100 % HBD only events in the absence of arabinose when Cas9 is present in the plasmids (p^HBD^ and p^HBD-ΔλRed^) indicates that a basal rate of recombination between the two direct repeats during replication in these situations is further enhanced compared to the plasmids which lack Cas9 (p^HBD-ΔCas9^ and p^HBD-ΔCas9-ΔλRed^) maybe by the low levels of Cas9 transcriptional leakage when Cas9 is present in those plasmids and by the slipped misalignment during DNA replication.

**Supplementary Table 1.** Plasmids used in this study

| **Plasmid** | **Relevant genotype or phenotype** | **Source** |
| --- | --- | --- |
| p^Pro-AG-i^  p^Pro-AG-ii^  p^Pro-AG-iii^  p^Pro-AG-Cas9 flipped-iv^  p^Pro-AG-v^  p^Pro-AG (SmR)^  p^ETag^  p^ETas^  p^Pro-AG (CmR)^  p^Pro-AG-ΔCas9^  p^Pro-AG-ΔλRed^  p^Nuc-cis^  p^Pro-MobV^  p^ETag+GFP-1^  p^ETg+GFP-1+100bpDR^  p^HBD^  p^HBD-ΔCas9^  p^HBD-ΔλRed^  p^HBD-ΔCas9- ΔλRed^  p^15A-λRed-Cas9^  p^SC101-λsgRNA1-^  ^λsgRNA2-GmR^  p^15A-λRed-Cas9-sgRNA-GFP-SmR^  λ-Control  λ-DPro-AG | Cm^R^, *ori*pSC101, low copy number vector expressing the AmpsgRNA (5’TTACTTCTGACAACGATCGG3’) from the *tet* promoter flanked with bla sequence homology arm 1 (HA1) and homology arm 2 (HA2), *Cas9* from tet promoter and λ*-Red* from pBAD promoter  p^Pro-AG-i^ derivative with *ori*p15A, low copy number vector  p^Pro-AG-i^ derivative with *ori*pBBR1, medium copy number vector  p^Pro-AG-iii^ derivative with flipped orientation of *Cas9* cassette  p^Pro-AG-iii^ derivative expressing *Cas9* and λ*-Red* from pBAD promoters  p^Pro-AG-iii^ derivative, expressing λ*-Red* and *Cas9* from pBAD promoter as a single operon and Sm^R^  Amp^R^, Gm^R^, *ori*F1, *lacI*+, *lacZα,* T7 promoter, *lac* operator, multicopy cloning and expression vector  p^ETag^ derivative, expressing Sm^R^ from the Amp promoter  p^Pro-AG-iii^, expressing λ*-Red* and *Cas9* from pBAD promoter as a single operon  p^Pro-AG (CmR)^ derivative with *Cas9* deletion  p^Pro-AG (CmR)^ derivative with λ*-Red* deletion  Gm^R^, *ori*pBBR1, conjugation machinery, *ori*T, expressing *Cas9* from pBAD promoter and sgRNA from tet promoter  Gm^R^, Cm^R^, *ori*pBBR1, conjugation machinery, *ori*T,  expressing AmpsgRNA from the *tet* promoter flanked with bla sequence HA1 and HA2, expressing λ*-Red* and *Cas9* from pBAD promoter as a single operon  Gm^R^, *ori*F1, *lacI*+, *lacZα,* T7 promoter, *lac* operator, out of frame GFP, multicopy cloning and expression vector  p^ETag+GFP-1^ derivative with 100 bp direct repeat  Sm^R^, *ori*p15A, low copy number vector expressing the GFPsgRNA2 (5’GAGCTGGACGGCGACGTAAA3’) from the *tet* promoter, *Cas9* from tet promoter and λ*-Red* from pBAD promoter  p^HBD^ derivative with *Cas9* deletion  p^HBD^ derivative with λ*-Red* deletion  p^HBD^ derivative with *Cas9* deletion and λ*-Red* deletion  Cm^R^, *ori*p15A, low copy number vector expressing λ*-Red* and *Cas9* from pBAD promoter  Gm^R^, *ori*pSC101, low copy number vector expressing the λ-sgRNA1(5’TACCGGACAATGAGTGACTG3’) from the *tet* promoter and the λ-sgRNA2 (5’AAGGCATTCCTACGAGCAGA3’) from the J23119 promoter  *ori*p15A, low copy number vector expressing the sgRNA-GFP (5’GAGCTGGACGGCGACGTAAA3’) from the *tet* promoter and Sm^R^ cassette flanked with phage sequence homology arm 1 (λ- HA1) and homology arm 2 (λ-HA2), expressing λ*-Red* and *Cas9* from pBAD promoter  Bacteriophage λ carrying constitutive non-targeting-sgRNA (GGTTTTGGACACTGGAACCG) and Sm^R^  Bacteriophage λ carrying constitutive sgRNA against GFP and Sm^R^ | This work  This work  This work  This work  This work  This work  Reference 1  This work  This work  This work  This work  Reference 5  This work  This work  This work  This work  This work  This work  This work  This work  This work  This work  This work  This work |

**
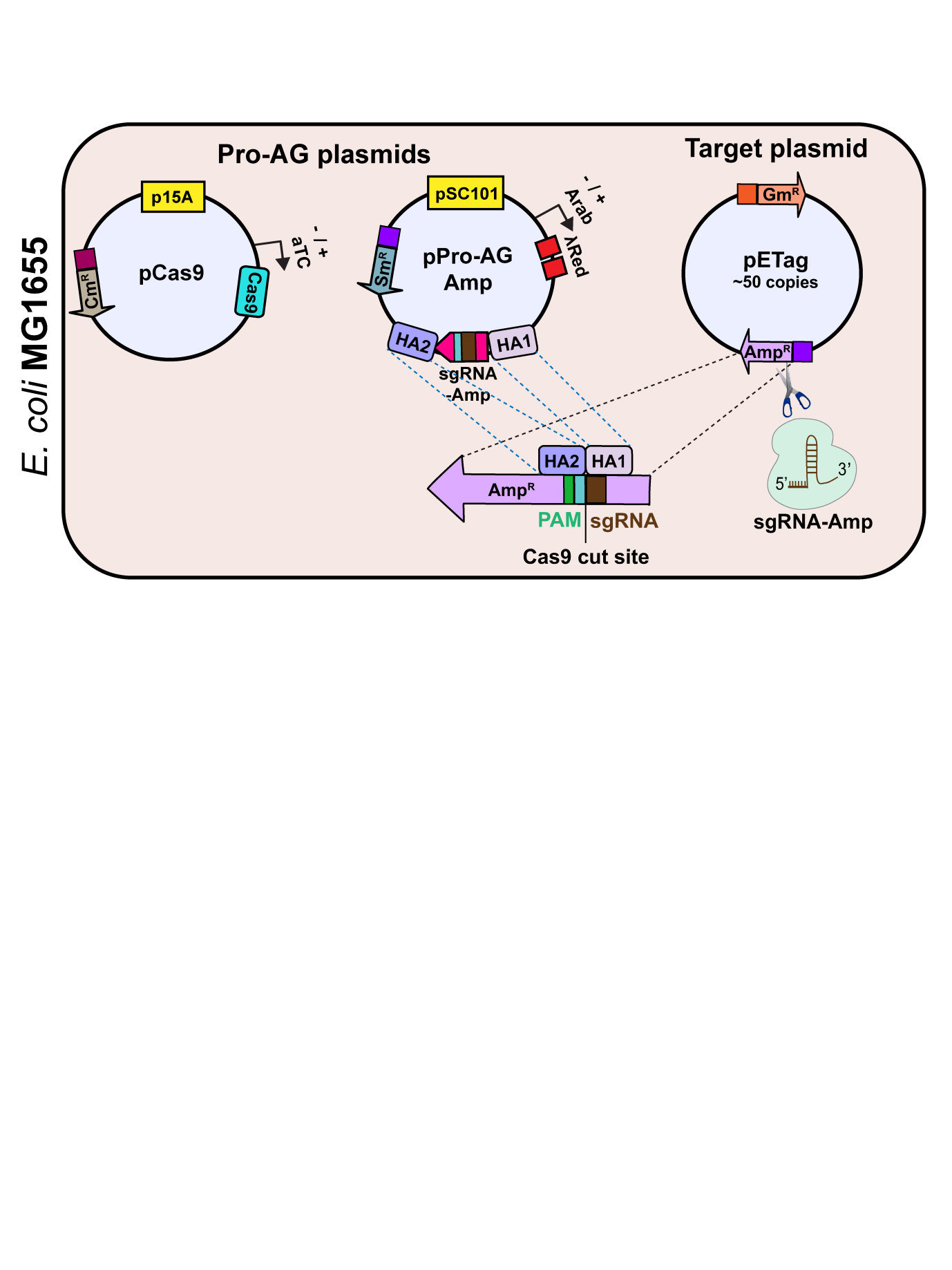
**

**Supplementary Figure 1. Schematic of Pro-AG study using three plasmids system.** In the original Pro-AG system, a sgRNA targeting a site in the beta-lactamase (*bla*) gene was flanked by sequences homologous to the cut site (homology arms: HA) and was launched from a low copy-number plasmid that also carried an arabinose-inducible λRed gene cassette, pPro-AG-Amp, which encodes three proteins that efficiently sustain homology-based repair of DNA double strand breaks (DSBs). The Cas9 transgene was carried on a separate low copy-number plasmid, pCas9, under conditional control of an aTC-inducible *tet* promoter. The *bla* target plasmid, pETag conferring resistance to ampicillin (Amp^R^) was present on a third high copy-number plasmid, which also bore a second marker for gentamicin resistance (Gm^R^). Inclusion of the Gm^R^ marker on the target plasmid permitted selection for gene-edited plasmids in which the *bla* gene was rendered non-functional by insertion of the sgRNA cassette. When bacteria carrying all three plasmids (the two Pro-AG encoding plasmids plus the target plasmid) were grown in the presence of inducers of both Cas9 and the λRed cassette, recovery of Amp^R^ colony forming unit (CFU) was reduced by ~5-logs. When these same ampicillin sensitive (Amp^S^) bacteria were grown on Gm plates, the number of CFU recovered fell by ~ 3-logs. All examined Amp^S^ Gm^R^ colonies generated by Pro-AG carried edited *bla* target genes with the sgRNA cassette inserted precisely into its cleavage site as expected. We attribute the difference between CFU recovery on Amp versus Gm selection plates to the effect of Pro-AG. The residual ~2-logs of reduced CFU (relative to non-induced controls) corresponded to the level of AR reduction achieved by simple cut-and-destroy CRISPR controls, which presumably also happens in the case of Pro-AG albeit at frequencies of <1 % relative to precise editing. Further analysis of the Pro-AG component on AR reduction revealed that it depends crucially on a self-amplifying process wherein levels of the sgRNAs expressed from a low copy number increase in a positive feedback cycle as the sgRNA cassette copies progressively into its high copy number target plasmid ^1^.

**
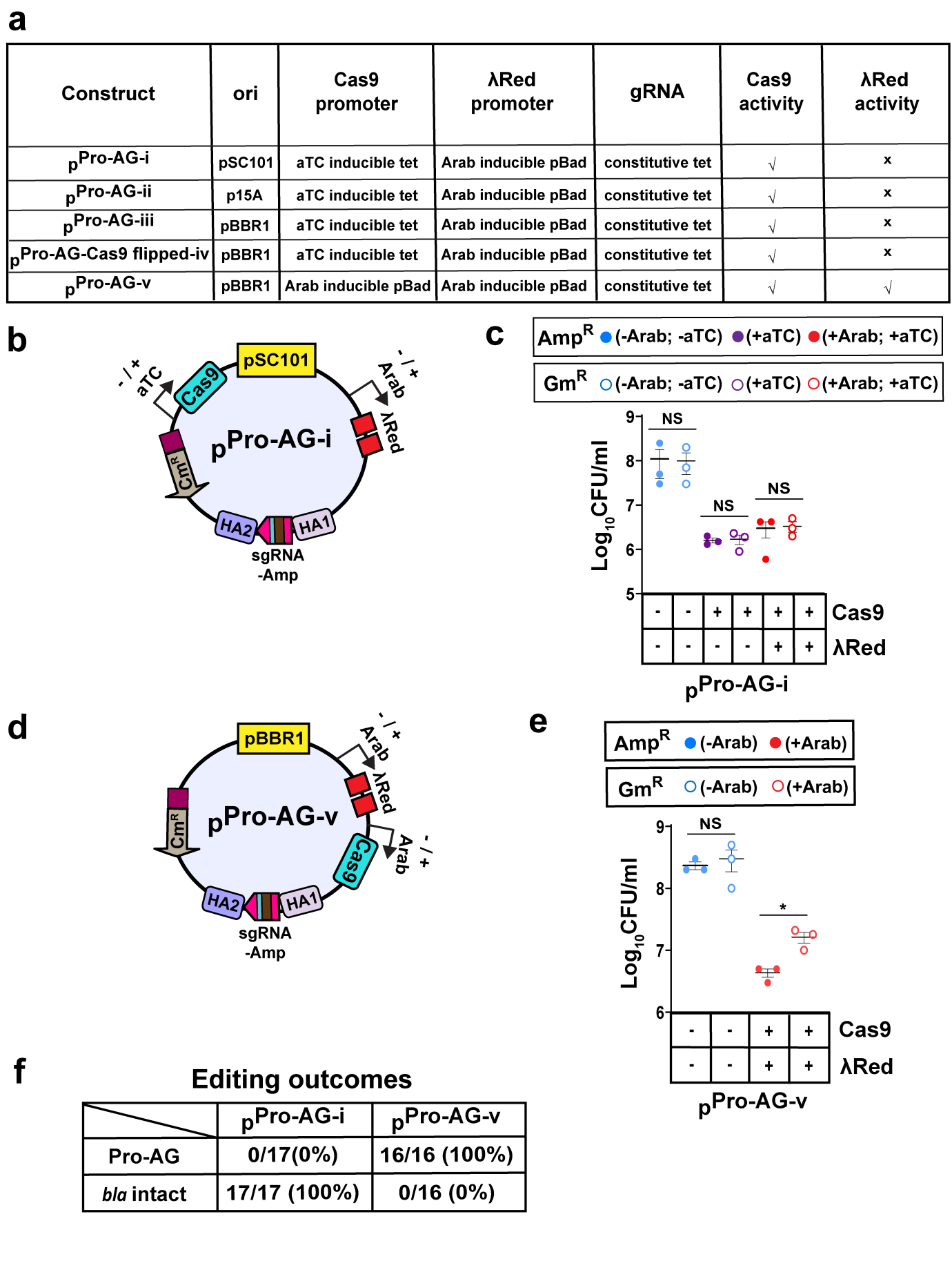
Supplementary Figure 2. p^Pro-AG^ plasmids configuration and editing events. a)** Table summarizing different Pro-AG constructs, their *ori,* types of promoters used to express Cas9, λRed, sgRNA-Amp. **b)** Schematic of p^Pro-AG-i^ construct.  **c)** Recovery of Amp^R^ or Gm^R^ CFU following Control (- aTC - arabinose, blue dots), Cas9 (+ aTC - arabinose, purple dots) and Pro-AG experiments (+ aTC + arabinose, red dots). **d)** Schematic of p^Pro-AG-v^ construct. **e)** Recovery of Amp^R^ or Gm^R^ CFU following Control (- arabinose, blue dots) and Pro-AG experiments (+ arabinose, red dots). **f)** DNA sequence analysis of plasmids isolated from single colonies from Gm plates after Pro-AG events (+ aTC + arabinose in p^Pro-AG-i^; and (+ arabinose in p^Pro-AG-v^). Data are plotted as the mean ± SEM, and analyzed by Student’s *t* test. N.S. = not significant (*P* > 0.05); **P* < 0.05.

**
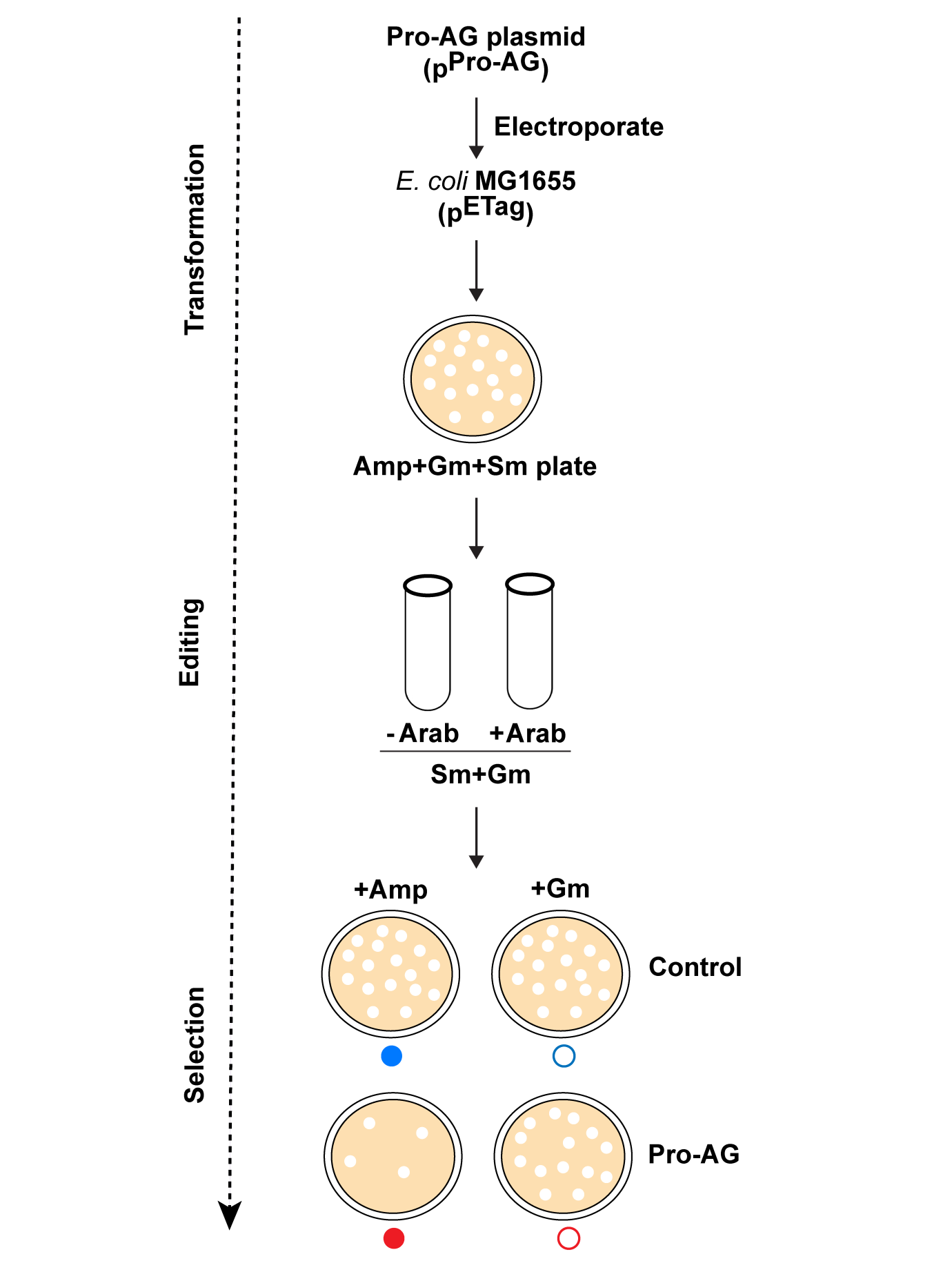
**

**Supplementary Figure 3. Pro-AG-mediated gene-editing.** Scheme used for editing *bla* gene from high copy number plasmid p^ETag^. *E. coli* MG1655 cells carrying p^ETag^ target plasmid carrying Amp resistance coding sequence were electroporated with donor plasmid (p^Pro-AG^) and grown overnight in LB agar plates with 0.2 % D-glucose, Amp, Gm, and Cm. Single colonies were then grown overnight in 5 mL LB in the absence (+ glucose, - arabinose, blue dots) or in the presence (+ glucose, + arabinose, red dots) of arabinose for λRed and Cas9 induction. Aliquots were diluted and plated on Amp or Gm plates for CFU enumeration. The CFU on Amp plates following Control (+ glucose, - arabinose) and Pro-AG (+ glucose, + arabinose) experiments are represented as solid blue dots and red dots while CFU on Gm plates following Control and Pro-AG experiments are represented as open blue dots and red dots.


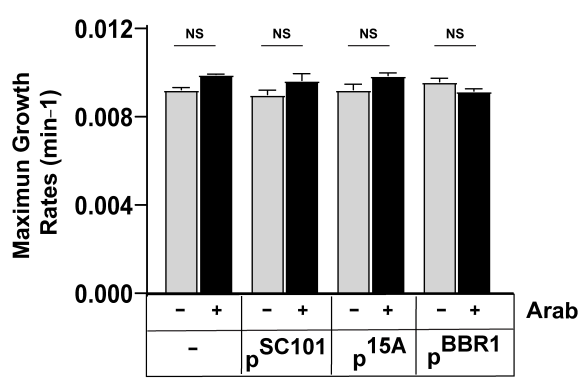

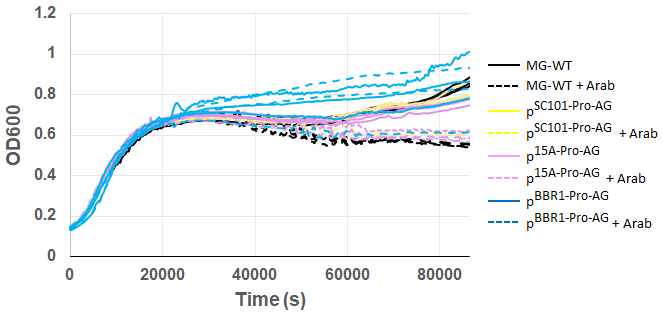


**b**

**a**

**Supplementary Figure 4. *E. coli* MG1655 cell growth is not affected with different copy number of λRed-Cas9. a)** MG-WT cells were electroporated with the Pro-AG plasmids (i) p^SC101-Pro-AG^, (ii) p^15A-Pro-AG^ or (iii) p^BBR1-Pro-AG^ carrying Sm resistance maker and plated overnight on Sm plates. Three colonies were picked from each plate and grown overnight in LB containing Sm with and without arabinose to acclimatize them to growth conditions. On the following day, 40 µl of these cultures were added to 4 ml LB containing Sm with and without arabinose and the growth was monitored for 24 hours and the optical densities at 600 nanometers (OD600) was observed every 5 minutes. MG-WT cells were used as internal control and were grown without Sm in all steps. **b)** Using a two-step analysis, we first identified the one-hour window in which the OD600 changed the most rapidly for each trial individually, and then the growth rates were calculated using the exponential growth equation over that period. The resulting maximal growth rate calculations were then used for analyses and plotted. Data are plotted as the mean ± SEM, and analyzed by Student’s *t* test. N.S. = not significant (*P* > 0.05).


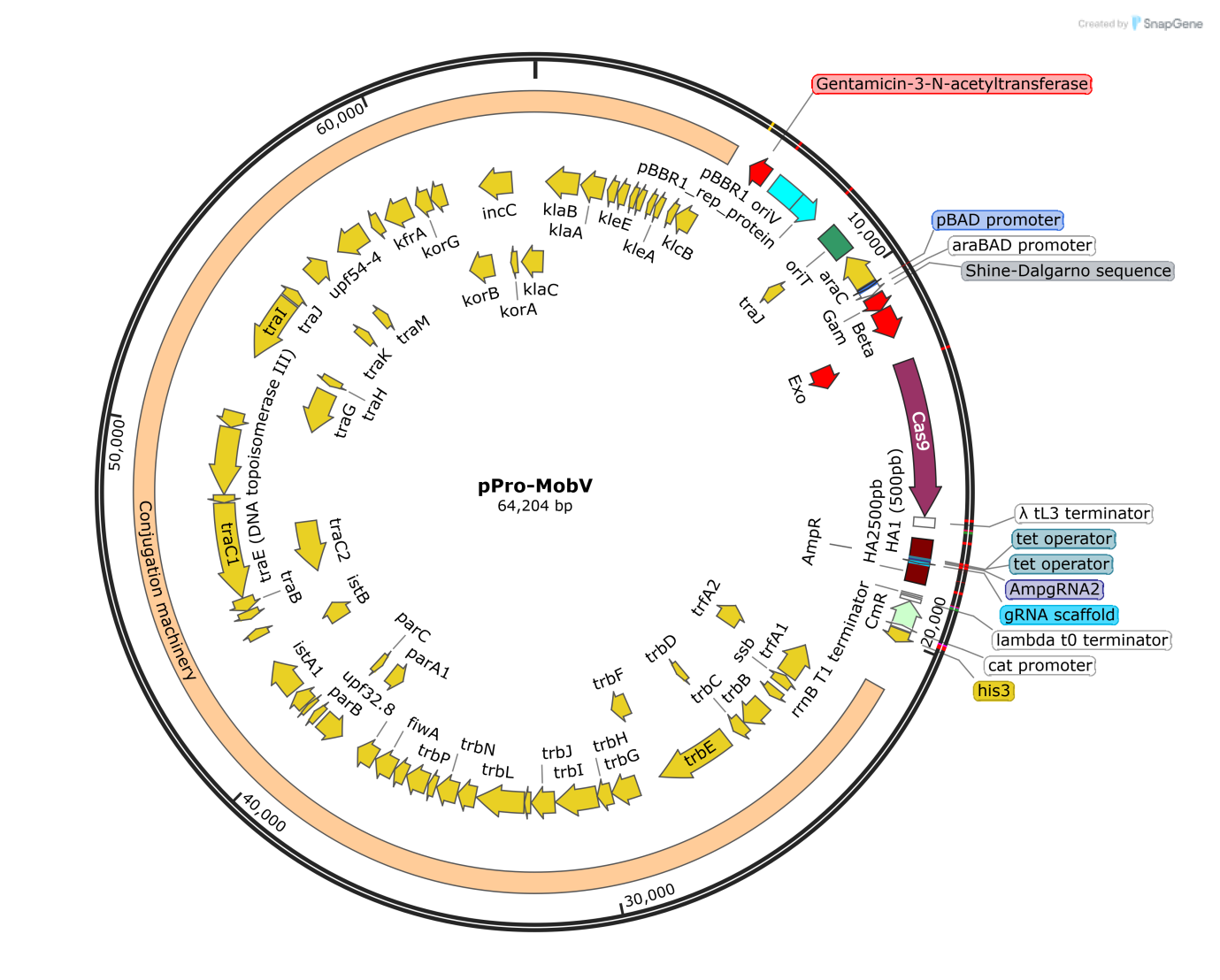
**Supplementary Figure 5. Plasmid map of p^ProMobV^.** The important features of p^Pro-MobV^ in detail like kil/kor regulon, Tra system, pBBR1 ori, Cas9 gene, λRed cassette, Cm and Gm resistance markers. The map was generated using Snapgene.

**a**


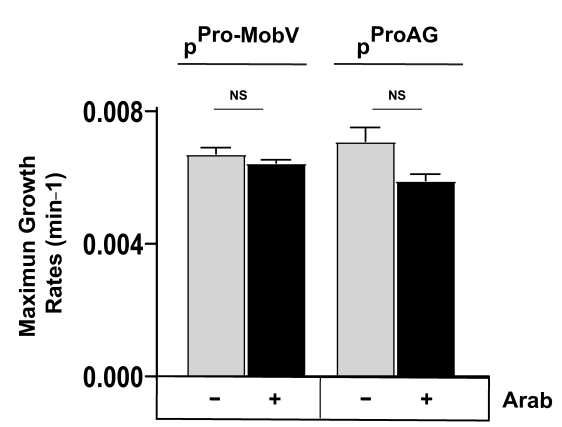

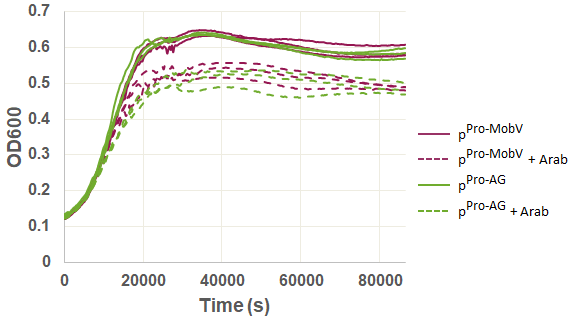


**b**

**a**

**a**

**Supplementary Figure 6. *E. coli* Epi300 cell growth is not affected with big size of p^Pro-MobV^. a)** Epi300 cell stock containing p^Pro-AG^ plasmid carrying Cm resistance maker or p^Pro-MobV^ carrying Gm and Cm resistance makers were streaked on Cm plates and grown overnight. Three colonies were picked from each plate and grown overnight in LB containing Cm with and without arabinose to acclimatize them to growth conditions. On the following day, 40 µl of these cultures were added to 4 ml LB containing Cm with and without arabinose and the growth was monitored for 24 hours and the optical densities at 600 nanometers (OD600) was observed every 5 minutes. b) Using a two-step analysis, we first identified the one-hour window in which the OD600 changed the most rapidly for each trial individually, and then the growth rates were calculated using the exponential growth equation over that period. The resulting maximal growth rate calculations were then used for analyses and plotted. Data are plotted as the mean ± SEM, and analyzed by Student’s *t* test. N.S. = not significant (*P* > 0.05).

**a**

**
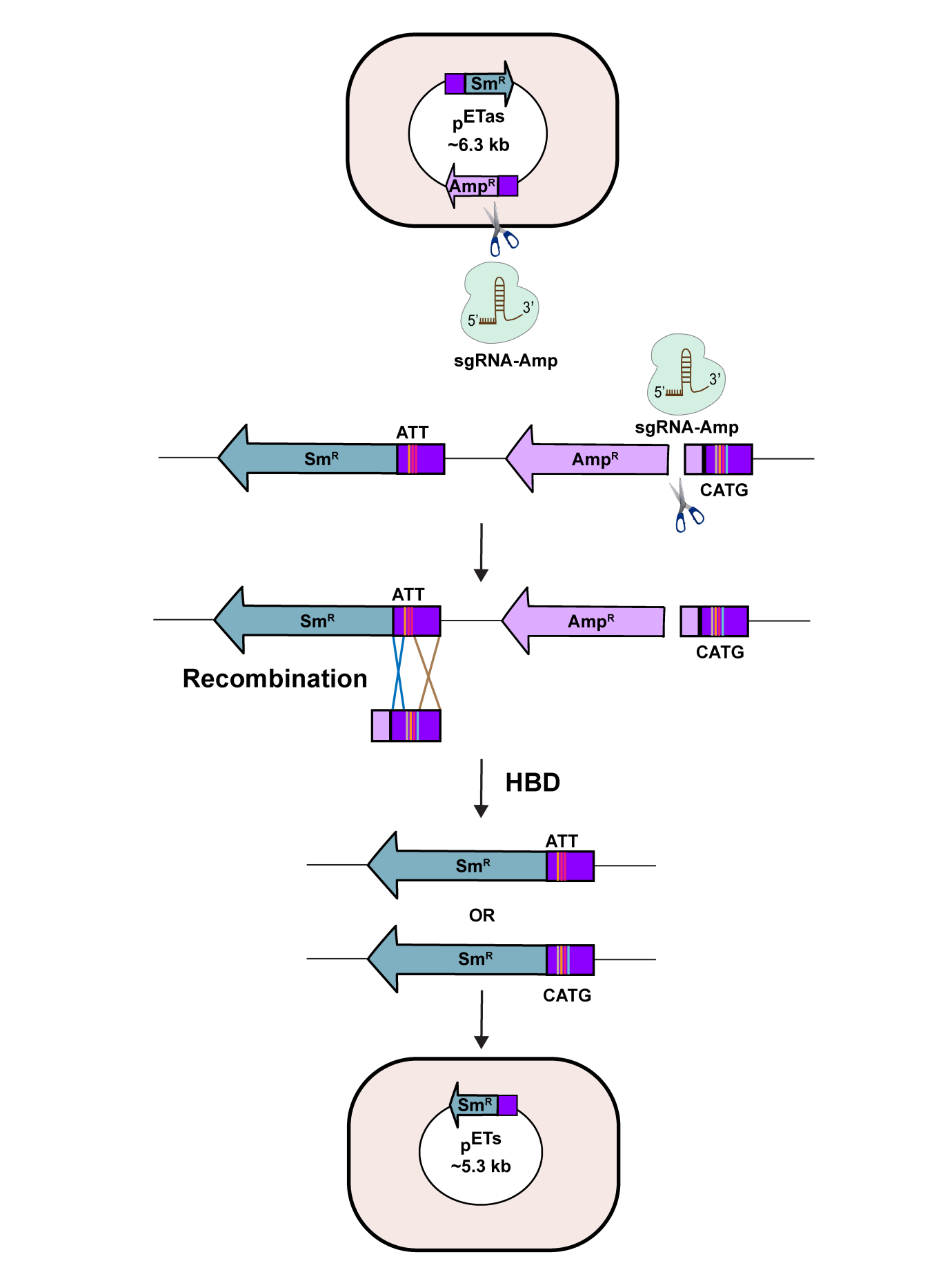
**

**Supplementary Figure 7. HBD is a homology dependent recombination event.** Schematic of p^ETas^ plasmid, the sgRNA-Amp and Cas9-mediated cut on Amp^R^ gene. The promoter regions are shown in purple with the three (ATT) and four (CATG) nucleotides that are different on each promoters. Recombination events between the two promoters are shown in blue and brown crosses. The resulting final p^ETs^ plasmid is shown at the bottom.

**
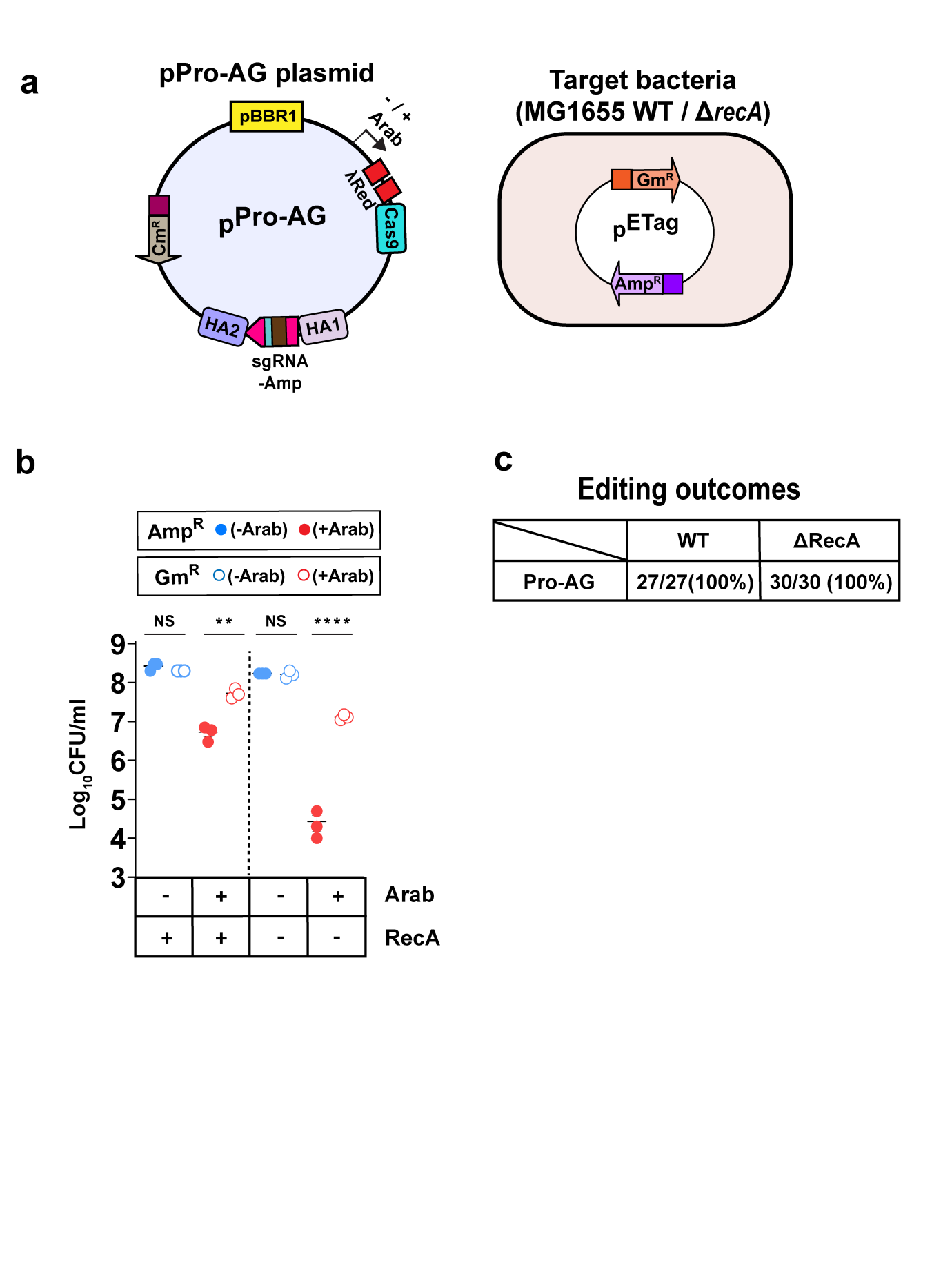
**

**Supplementary Figure 8. Efficient reduction of Amp^R^ CFU and recovery on Gm plates in *recA* mutant target cells. a)** Schematic of p^Pro-AG^ plasmid and p^ETag^ plasmid. **b)** Comparison of CFU following Control and Pro-AG in *E. coli* MG1655 WT or Δ*recA* target cells containing p^ETag^ plasmid. **c)** DNA sequence analysis of target plasmids isolated from single colonies from Gm plates after Pro-AG events. Data are plotted as the mean ± SEM, and analyzed by Student’s *t* test. N.S. = not significant (*P* > 0.05); ***P* < 0.01; ****P < 0.0001.

**
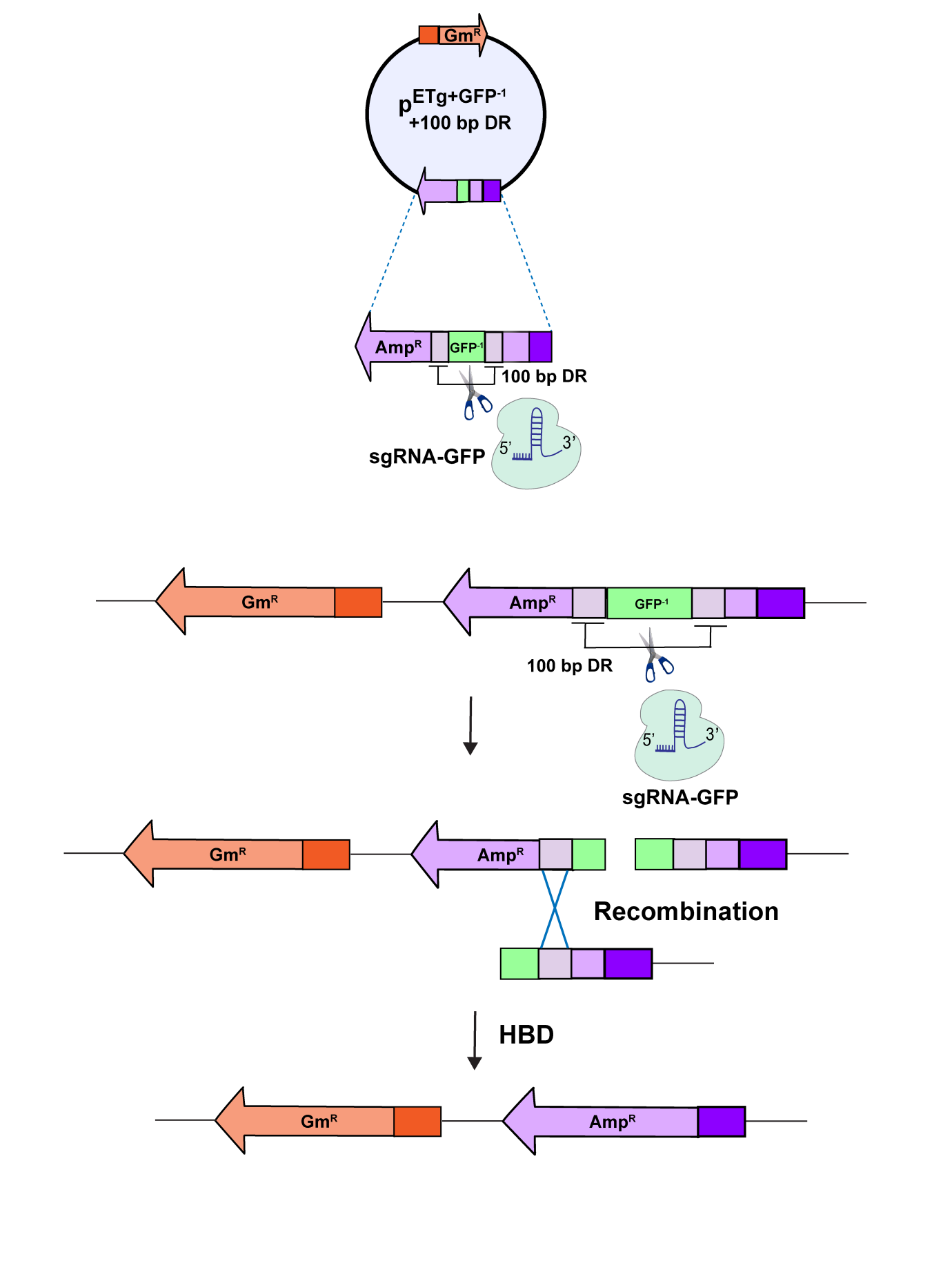
Supplementary Figure 9. HBD precisely reverts back ampicillin resistance.** Schematic of p^ETg+GFP-1+100bpDR^ plasmid, the sgRNA-GFP and Cas9-mediated cut on GFP cassette inserted on Amp^R^ gene. The 100 bp repeat regions are shown in light purple and are flanking the GFP cassette. A recombination event between the two repeat regions is shown as blue cross. Formation of the final p^ETag^ plasmid is shown at the bottom.

**
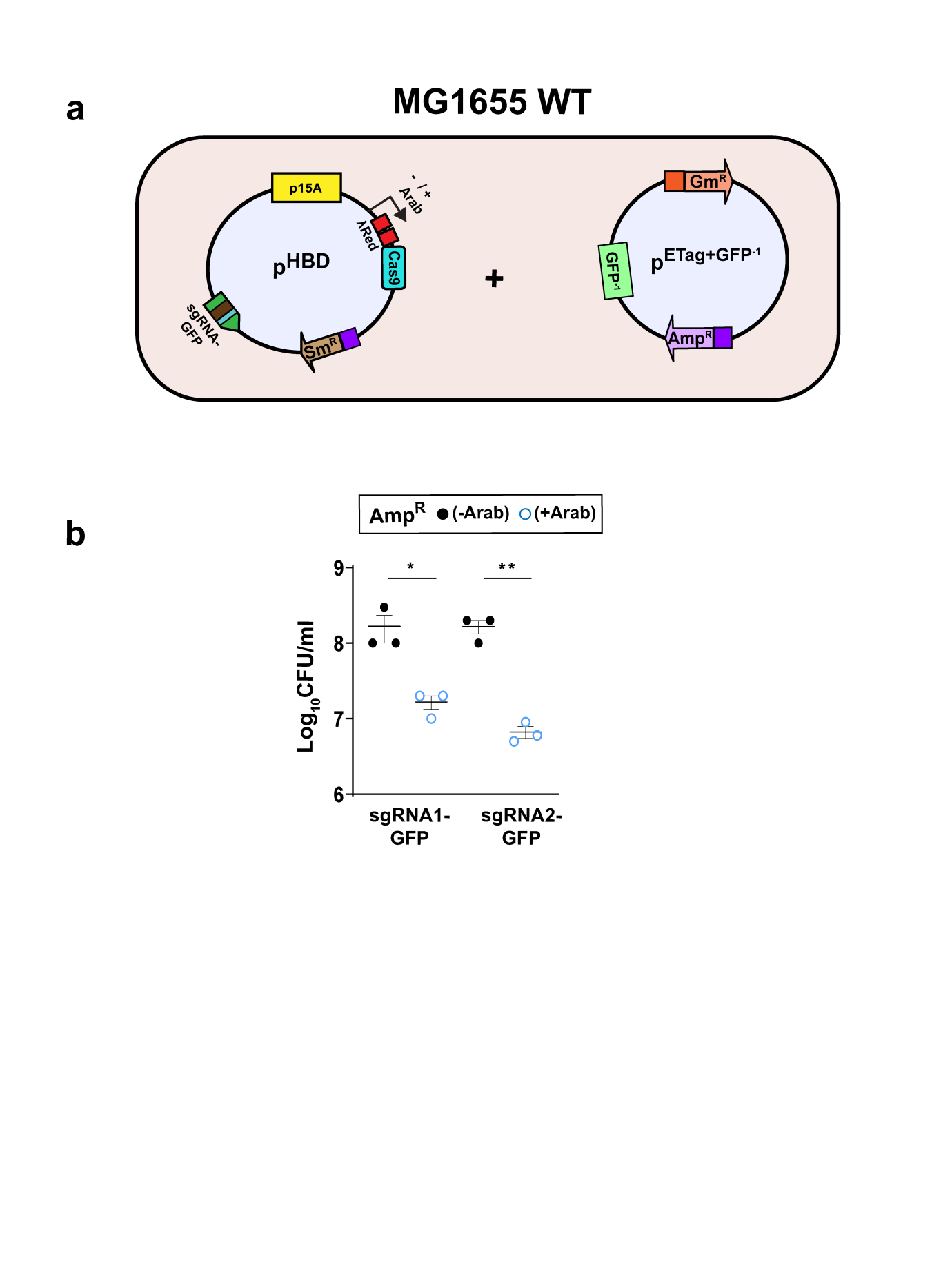
Supplementary Figure 10. Target cleavage efficiency of the sgRNAs. a)** Schematic of the p^HBD^ plasmid mediated editing of *gfp* target encoded on a high copy number p^ETag+GFP-1^ plasmid in *E. coli* MG1655. **b)** *E. coli* MG1655 cells carrying p^ETag+GFP-1^ target plasmid encoding Amp and Gm resistance were electroporated with the pHBD plasmid carrying sgRNA1-GFP or sgRNA2-GFP and grown overnight in LB agar plates with Amp and Sm. Single colonies were then grown over night in 5 mL LB in the absence or in the presence of arabinose for λRed and Cas9 induction. Aliquots were diluted and plated on Amp plates for CFU enumeration. The CFU on Amp plates following Control (- arabinose, solid dots) and HBD (+ arabinose, open dots) experiments are represented as solid black dots and open blue dots. Data are plotted as the mean ± SEM, analyzed by Student’s *t* test. **P* < 0.1; **P < 0.01.

**
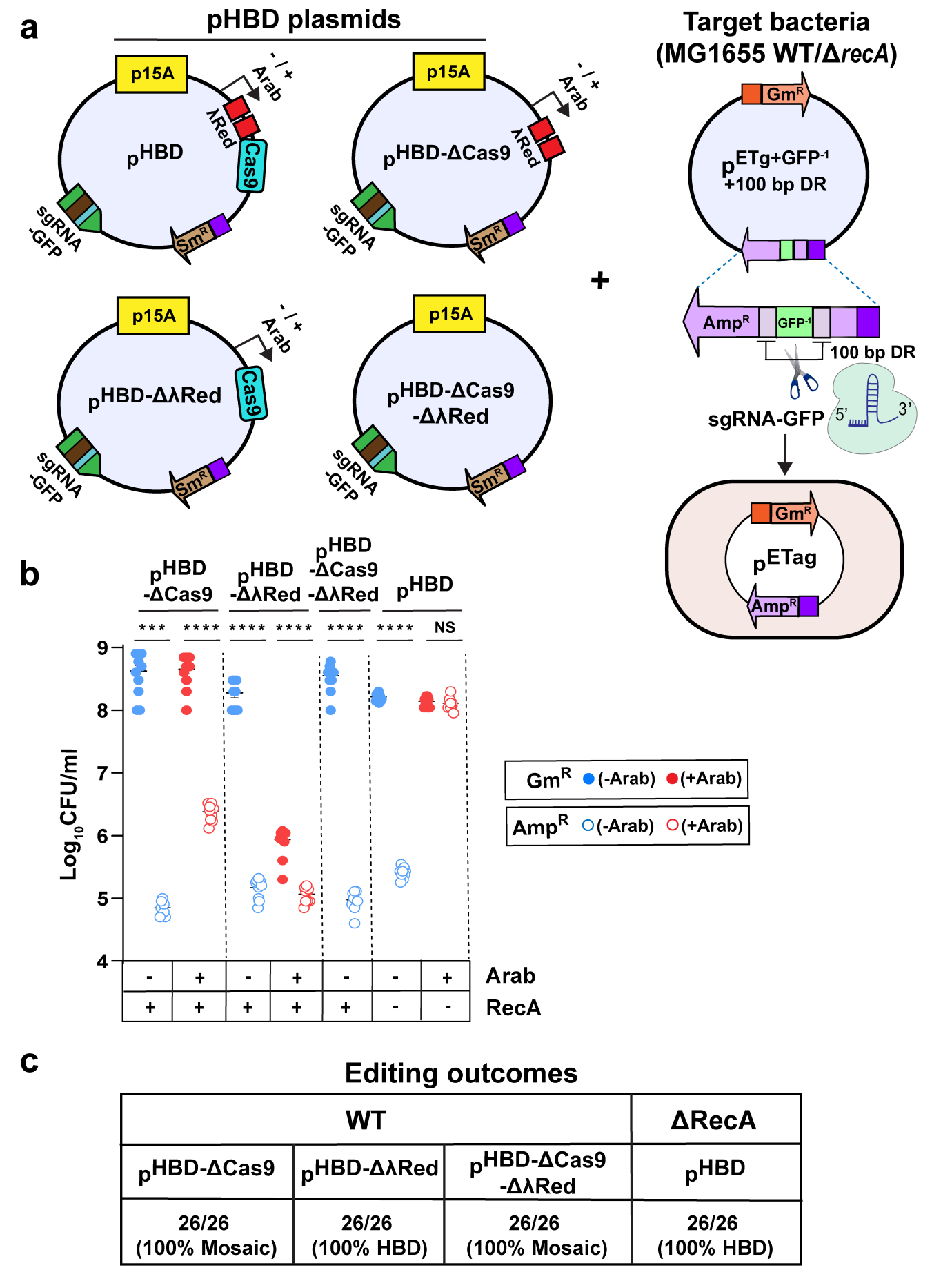
**

**Supplementary Figure 11. Efficient recovery of Amp^R^ colonies using HBD recombination. a)** Schematic of p^HBD^, p^HBD-ΔCas9^ (Cas9 deleted version of pHBD), p^HBD-ΔλRed^ (λRed deleted version of p^HBD^) and p^HBD-ΔλRed-ΔCas9^ (λRed and Cas9 deleted version of pHBD) plasmids (left), p^ETg+GFP-1+100bpDR^ and p^ETag^ plasmid (right). **b)** Comparison of CFU in the presence or absence of arabinose on Amp or Gm plates in either WT or Δ*recA* target cells carrying p^ETg+GFP-1+100bp^ DR target plasmid. **c)** DNA sequence analysis of target plasmids isolated from single colonies from Amp plates in the presence of arabinose. Data are plotted as the mean ± SEM, representing three independent experiments performed in triplicate and analyzed by Student’s *t* test. N.S. = not significant (*P* > 0.05); ****P* < 0.001; ****P < 0.0001.

**
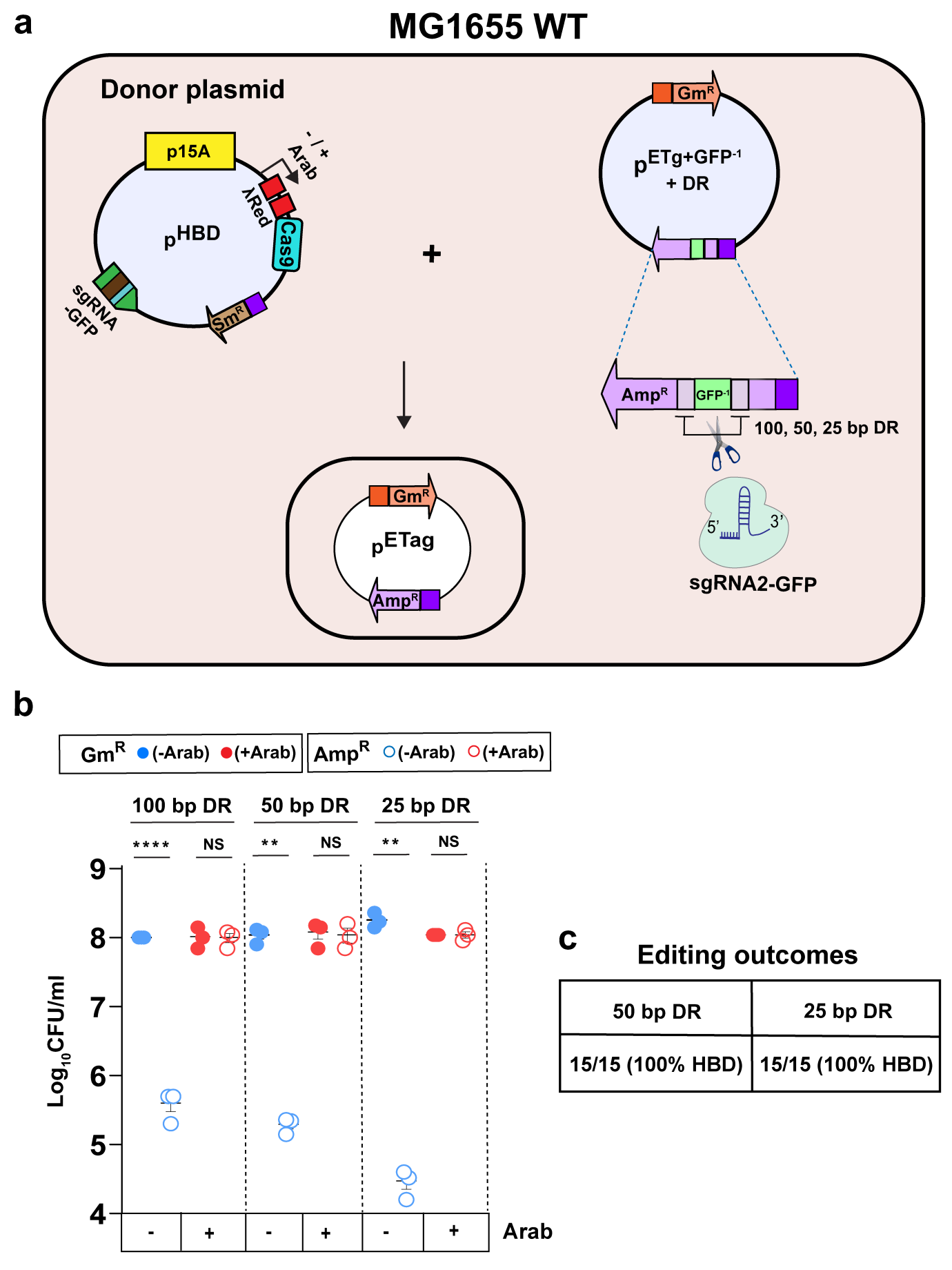
**

**Supplementary Figure 12. Basal HBD depends on the length of the direct repeats. a)** Schematic of the p^HBD^ plasmid mediated editing of *gfp* target encoded on a high copy number p^ETg+GFP-1+DR^ plasmid carrying (p^ETg+GFP-1+100bpDR^, p^ETg+GFP-1+50bpDR^ or p^ETg+GFP-1+25bpDR^) in *E. coli* MG1655. **b)** Comparison of CFU in the presence or absence of arabinose on Amp or Gm plates. **c)** DNA sequence analysis of target site from single colonies isolated from Amp plates following arabinose addition for 50 bp and 25 bp direct repeat containing receiver plasmids. Data are plotted as the mean ± SEM, analyzed by Student’s *t* test. **P* < 0.1; **P < 0.01. N.S. = not significant (*P* > 0.05); ***P* < 0.01; ****P < 0.0001.

**
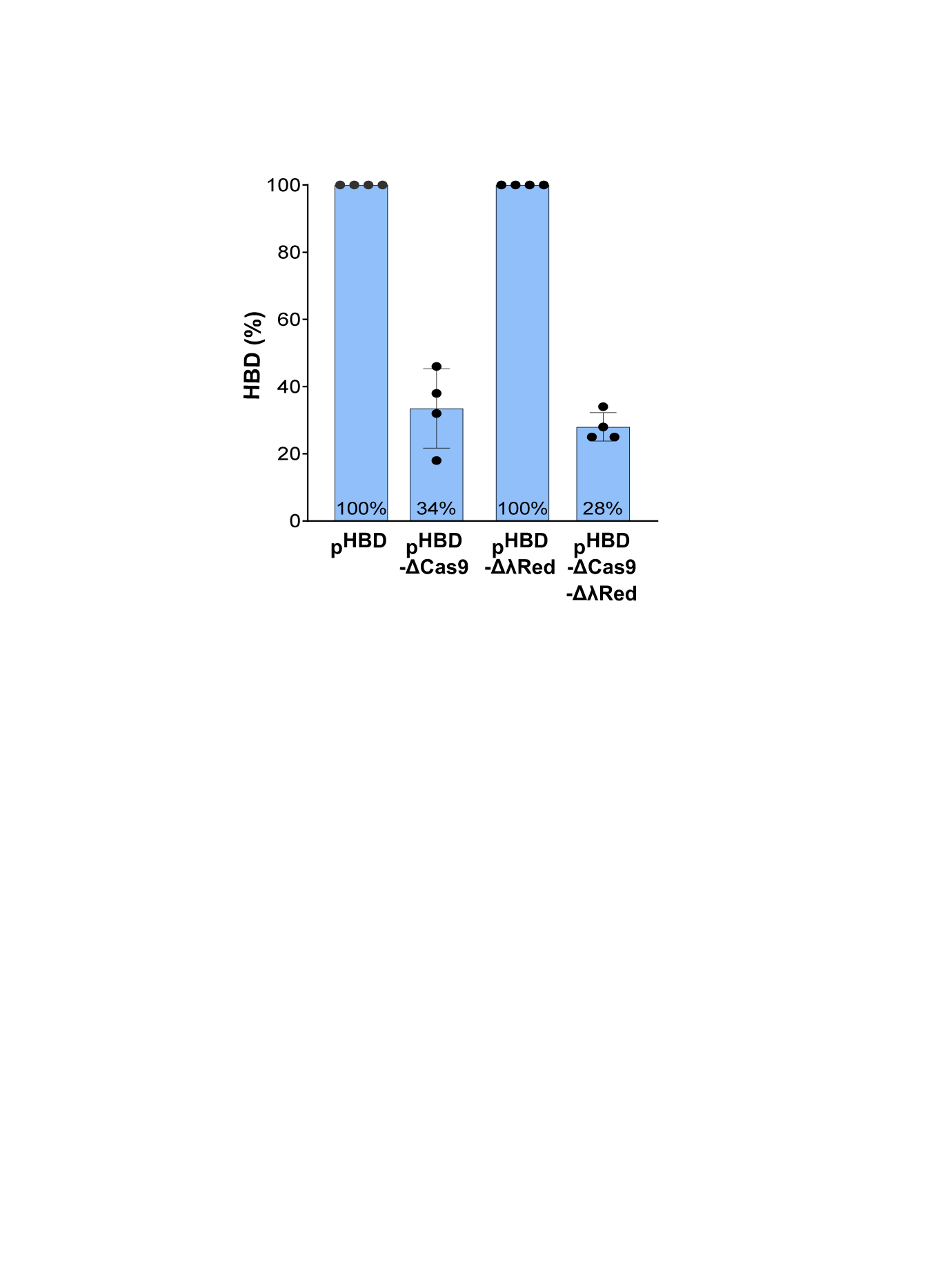
Supplementary Figure 13. Sequence analysis of mosaic reads.** Graphical representation of basal HBD events at target site in p^ETg+GFP-1+100bpDR^ plasmid in the absence of arabinose when p^HBD^ and the variant plasmids p^HBD-ΔCas9^, p^HBD-ΔλRed^ and p^HBD-ΔCas9-ΔλRed^ plasmids were used.

**
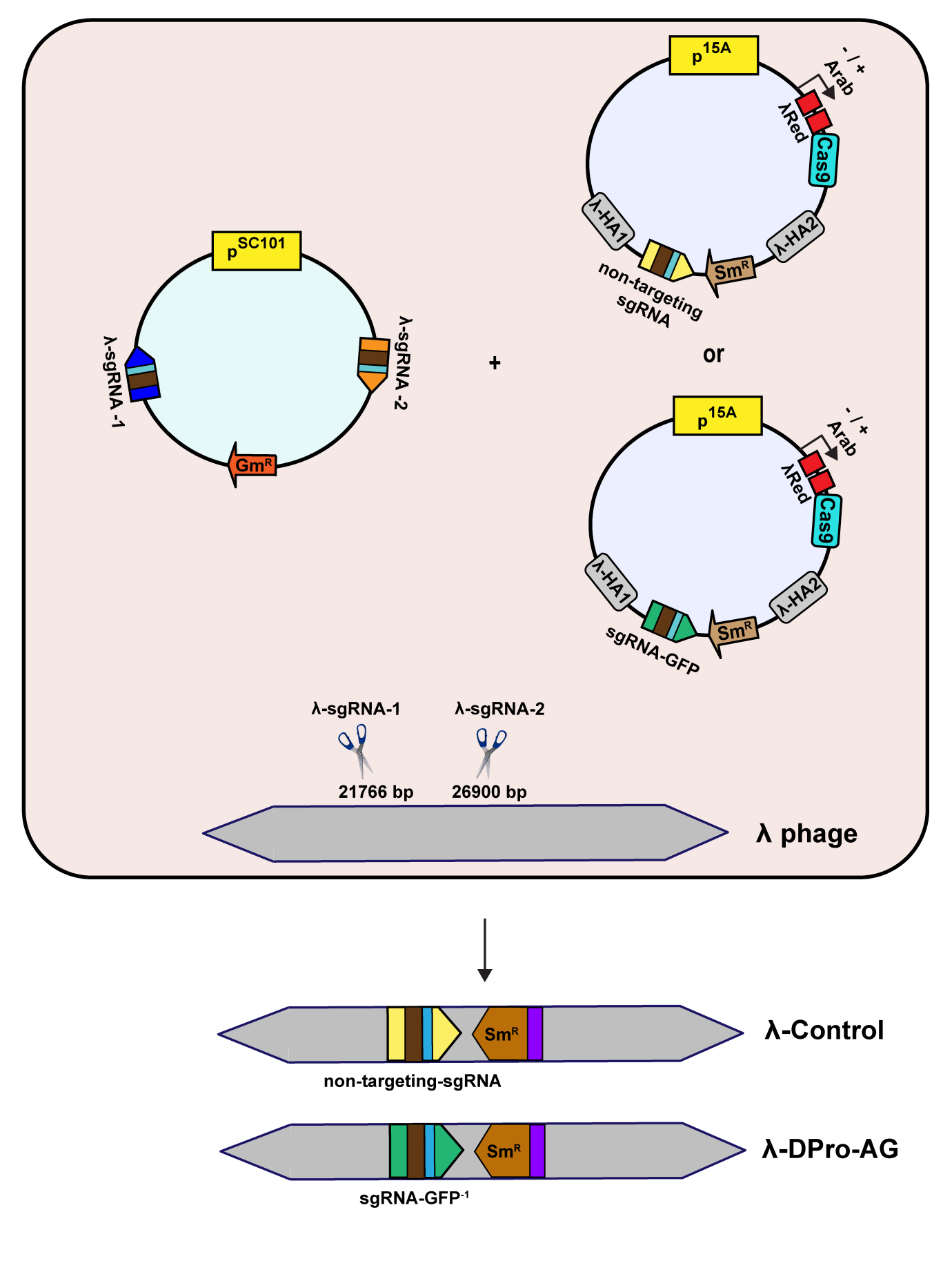
**

**Supplementary Figure 14. Generation of λ-DPro-AG.** *E. coli* MG1655 cells carrying λ-lysogens were electroporated with the p^SC101^ plasmid that carries two constitutive sgRNAs (constitutive tet promoter to express the sgRNA1 and constitutive J23119 promoter to express the sgRNA2) to delete a region of the λ genome spanning from 21,766 bp to 26,900 bp and p^15A-λRed-Cas9-non-targeting-sgRNA-SmR^ or p^15A-λRed-Cas9-sgRNA-GFP-SmR^ plasmid that carries arabinose inducible λRed and Cas9, constitutive tet promoter to express non-targeting sgRNA or sgRNA-GFP and Sm^R^ gene cassette flanked by homology arms. Bacterial cells were then plated on LB agar plates with Gm+Sm and grown overnight at 30 °C. Single colonies were then grown overnight in 5 mL LB with Gm+Sm+arabinose and plated on Sm plates for selecting the edited λ phage (λ-Control or λ-DPro-AG) and insertion of non-targeting-sgRNA (i.e., which matches no sequences present in either the target plasmid or bacterial genome) or sgRNA-GFP and Sm^R^ cassettes into phage genome was sequence verified.

**References**

1. Valderrama, J.A., Kulkarni, S.S., Nizet, V. & Bier, E. A bacterial gene-drive system efficiently edits and inactivates a high copy number antibiotic resistance locus. *Nature Communications* **10**, 5726 (2019).

2. Feschenko, V.V. & Lovett, S.T. Slipped misalignment mechanisms of deletion formation: analysis of deletion endpoints. *J Mol Biol* **276**, 559-569 (1998).

3. Bi, X. & Liu, L.F. recA-independent and recA-dependent intramolecular plasmid recombination. Differential homology requirement and distance effect. *J Mol Biol* **235**, 414-423 (1994).

4. Bi, X. & Liu, L.F. A replicational model for DNA recombination between direct repeats. *J Mol Biol* **256**, 849-858 (1996).

5. Hamilton, T.A. et al. Efficient inter-species conjugative transfer of a CRISPR nuclease for targeted bacterial killing. *Nature Communications* **10**, 4544 (2019).
